# Supramolecular Polymerization as a Tool to Reveal the Magnetic Transition Dipole Moment of Heptazines

**DOI:** 10.1021/jacs.4c02174

**Published:** 2024-05-30

**Authors:** Fan Xu, Hao Su, Joost J. B. van der Tol, Stef A. H. Jansen, Youxin Fu, Giulia Lavarda, Ghislaine Vantomme, Stefan Meskers, E. W. Meijer

**Affiliations:** † Institute for Complex Molecular Systems and Laboratory of Macromolecular and Organic Chemistry, Eindhoven University of Technology, Eindhoven 5600 MB, Netherlands; ‡ College of Polymer Science and Engineering and State Key Laboratory of Polymer Materials Engineering, Sichuan University, Chengdu 610065, China; § Stratingh Institute for Chemistry, University of Groningen, Nijenborgh4, Groningen 9747AG, Netherlands; ∥ Institute for Complex Molecular Systems and Molecular Materials and Nanosystems, Eindhoven University of Technology, Eindhoven 5600 MB, Netherlands; ⊥ School of Chemistry and RNA Institute, UNSW, Sydney NSW 2052, Australia

## Abstract

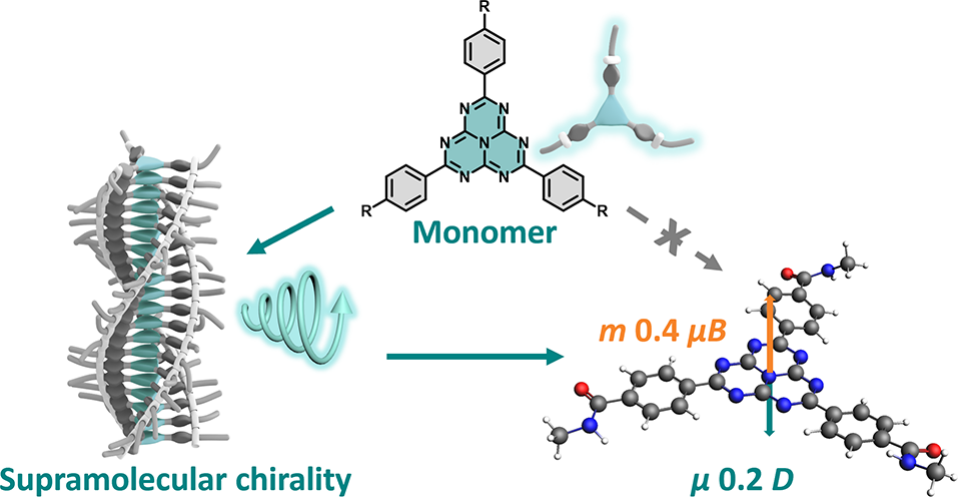

Heptazine derivatives have attracted significant interest due to their small S_1_-T_1_ gap, which contributes to their unique electronic and optical properties. However, the nature of the lowest excited state remains ambiguous. In the present study, we characterize the lowest optical transition of heptazine by its magnetic transition dipole moment. To measure the magnetic transition dipole moment, the flat heptazine must be chiroptically active, which is difficult to achieve for single heptazine molecules. Therefore, we used supramolecular polymerization as an approach to make homochiral stacks of heptazine derivatives. Upon formation of the supramolecular polymers, the preferred helical stacking of heptazine introduces circular polarization of absorption and fluorescence. The magnetic transition dipole moments for the S_1_ ← S_0_ and S_1_ → S_0_ are determined to be 0.35 and 0.36 Bohr magneton, respectively. These high values of magnetic transition dipole moments support the intramolecular charge transfer nature of the lowest excited state from nitrogen to carbon in heptazine and further confirm the degeneracy of S_1_ and T_1_.

## Introduction

In organic molecular materials, the mean free path of an electron is usually severely limited, and its angular momentum is heavily quenched.^1^ Quantum Hall effects, topologically protected conduction states, and other quantum conduction phenomena are practically impossible to realize with organics. The limited size of the molecules, strong coupling with vibrations, and an abundance of crystal defects are to blame. To improve electron delocalization, there is a global effort to design and synthesize molecules with monodisperse mass distribution and extended conjugation.^2−7^

Among extended conjugated carbon nitrides, heptazine has garnered significant attention in recent years owing to its unique electronic and optical properties, for instance, thermally activated delayed fluorescence (TADF).^8,9^ By designing specific structures, inverted singlet and triplet excited states are proposed, which breaks Hund’s rule.^10,11^ Furthermore, heptazine derivatives find applications in various fields, for instance, photocatalysis of water splitting.^8,12−15^ These unique electronic and optical properties are closely related to its lowest excited state.^8−14^ However, the precise nature of the lowest excited state of heptazine remains unclear.

The magnetic transition dipole is among the first-order terms in a series expansion of the interaction of the molecule underdoing the transition with the electromagnetic field.^16^ As a result, it reflects the nonlocal response of the molecules that is expected when the coherence length of the wavefunction is no longer negligible compared to the wavelength of light. The magnetic transition dipole moment (***m***) describes the circular component of the change transfer, indicating the rotation of electrons during the transition; thus, is crucial for unveiling the nature of the lowest excited state.^17^ In a truly breathtaking development over the last few years, improvements in synthesis have resulted in molecules with chromophoric centers containing sp^2^ hybridized atoms with high ***m***.^18−31^ These largely conjugated chromophores are intrinsically chiral and are the result of the impressive synthesis and separation of enantiomers. The magnetic transition dipole moment of the enantiomers of chiral molecules can be quantitatively determined from circular dichroism spectroscopy. To date, the determination of the magnetic transition dipole moments of molecules with flat π systems remains challenging, especially for heptazine, which presents a synthetic challenge when attempting to generate chiral variants.

Supramolecular chirality, which refers to the nonsymmetric arrangement of molecules within a noncovalent system,^32,33^ emerges as one of the most promising approaches for chiroptoelectronic^34^ and spintronics.^35−38^ Supramolecular polymers, as one-dimensionally ordered assemblies,^39,40^ are one of the best systems for introducing supramolecular chirality.^41−43^ They are formed by noncovalent bonding and therefore have significant advantages in terms of dynamics and pathway complexity.^44−50^ Their high degree of tunability and versatility provides a valuable complement to traditional covalent polymers and contributes to the creation of adaptive systems and innovative materials.^51−56^

**Figure 1:**
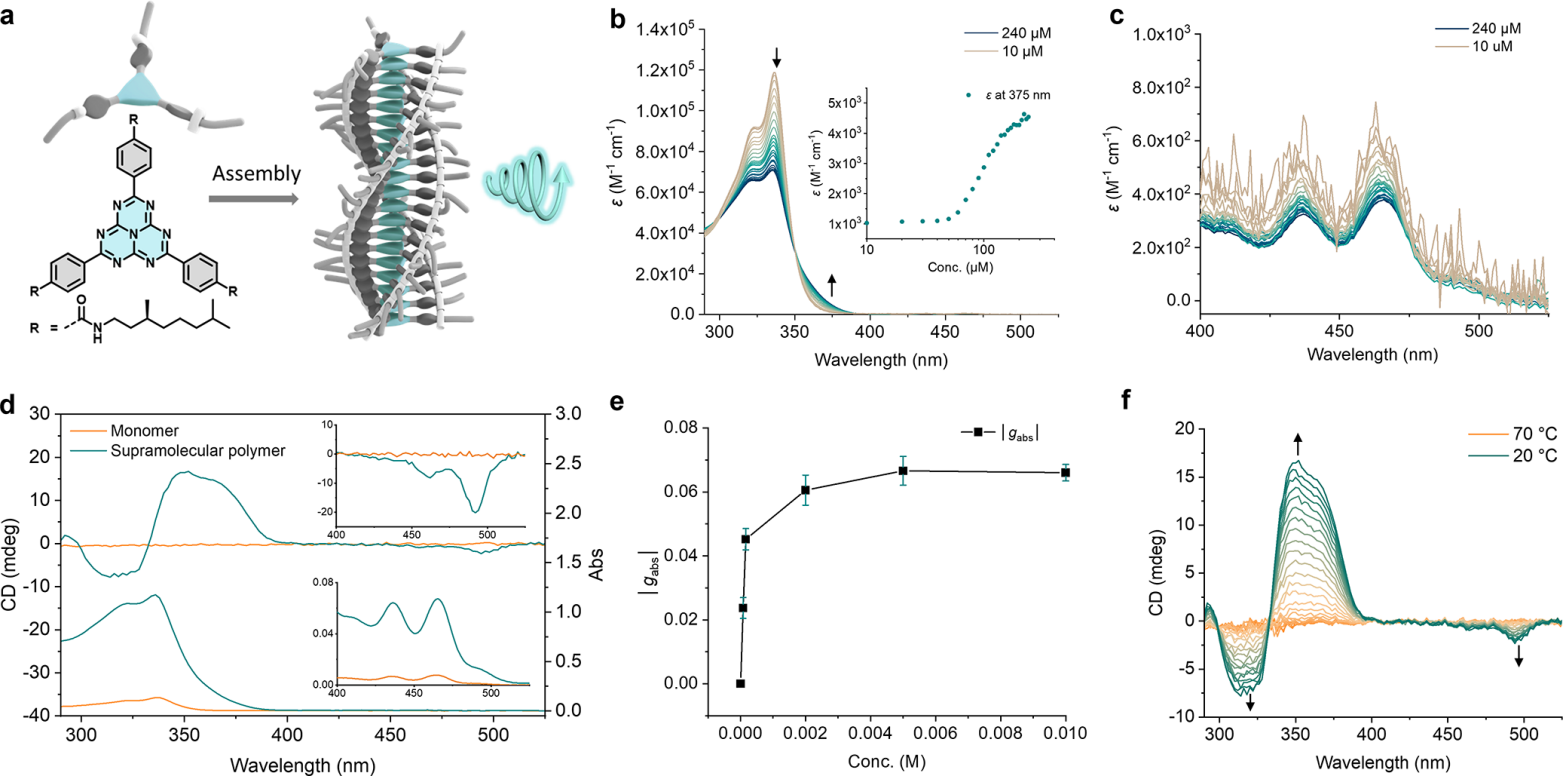
(a) Molecular structure of *S*-**H** and cartoon of a supramolecular polymer of *S*-**H** with circularly polarized luminescence (CPL) (b) Concentration-dependent UV–vis absorption spectra of *S*-**H** (10–240 μM) in toluene, inset: ε at 375 nm of *S*-**H** in toluene at different concentrations, and (c) enlarged spectra between 400 and 525 nm. (d) CD and UV–vis absorption of toluene solution of *S*-**H** in the 1 mm cuvettes at 10 and 160 μM, separately. Inset: CD and UV–vis absorption spectra at the region of 400–525 nm. (To increase the resolution of spectra at the 400–525 nm region, the samples were measured in a 1 cm cuvette). (e) Values of dissymmetry factor of absorption (|*g*_abs_|) of *S*-**H** in toluene at different concentrations. (f) CD spectra of a toluene solution of *S*-**H** (160 μM) upon cooling with a rate of −2 K min^–1^.

In the present study, we show that supramolecular polymerization can induce a high circular polarization in the optical transition between the ground and the lowest excited singlet state and thus reveal the spatial coherence of the electronic states of molecules. The achiral heptazine core was substituted with chiral alkyl chains, which offer a chiral bias to the helical stacking of heptazine (*S***-H**) (Figure 1a). The resulting supramolecular polymer exhibits circularly polarized absorption and fluorescence with high dissymmetric factors. The dissymmetric factor and quantum yield of the solution can be controlled by altering the ratio of the solvent mixture. Notably, the resulting high degree of circular polarization in the optical transition between the ground and lowest excited singlet state offers a method to characterize the magnetic transition dipole moment through supramolecular polymerization.

## Results and Discussions

The synthesis of *S***-H** is detailed in the Supporting Information. The assembly behavior of *S***-H** was studied in toluene by using circular dichroism (CD), UV–vis absorption, ^1^H NMR, and FT-IR spectroscopy. The absorption spectrum of monomeric dissolved heptazine shows a strong S_n_ ← S_0_ absorption band at 300–375 nm and a very weak S_1_ ← S_0_ absorption band at 420–515 nm with molar extinction coefficients (ε) of 1.2 × 10^5^ and 5 × 10^2^ M^–1^ cm^–1^, respectively, which is comparable with other heptazine derivatives (Figure 1b,c).^8^ Concentration-dependent UV–vis spectroscopy measurements in toluene revealed a broadening of the absorption band of S_n_ ← S_0_ with a clear isosbestic point at 351 nm (Figure 1b) due to aggregation, while the shape of the band of S_1_ ← S_0_ remained consistent (Figure 1c). The critical aggregation concentration (CAC) of approximately 50 μM was determined based on the sharp transition observed in the curve of absorption as a function of concentration (Figure 1b). The formation of one-dimensional aggregates was confirmed by the atomic force microscopy (AFM) studies, in which the diameter of fibers is around 1.2 nm (Figure S1). This diameter closely corresponds to the distance between the center N atom in the heptazine core and the terminal C atom of the alkyl chain in the molecule.

Above the CAC, toluene solutions of *S***-H** exhibit CD signals at both bands (Figure 1d), suggesting the formation of chiral supramolecular polymers. Changes in the CD signal and UV–vis absorption spectra can be reversibly controlled by heating and cooling (Figures 1f and S2). Plotting the CD signal at 360 nm against different temperatures results in a nonsigmoidal curve with a sharp transition at *T*_*e*_ (Figure S3). To analyze the cooling curves of *S***-H** at different concentrations, we employed a thermodynamic mass-balance model (details in SI). The fitting results revealed that the supramolecular polymerization of *S***-H** follows a cooperative mechanism, characterized by an elongation enthalpy *ΔH*_*e*_ of −49.2 kJ mol^–1^, an entropy *ΔS* of −84.0 J mol^–1^ K^–1^, and a nucleation penalty (NP) of 10.2 kJ mol^–1^ (Table S1). We further measured the FT-IR spectra of *S***-H** in toluene, which indicated the presence of hydrogen-bonded species in the solution, as evidenced by the N–H stretching vibration at 3257 cm^–1^ and the C=O stretching vibration at 1625 cm^–1^ (Figure S4). Temperature-dependent ^1^H NMR spectra revealed the upfield shifts of aromatic protons (H^a^ and H^b^) upon cooling, suggesting the occurrence of π–π stacking among these phenyl groups (Figure S5). Therefore, the heptazine monomers are stacked on top of each other through π–π stacking and hydrogen bonding to form homochiral supramolecular polymers, following a cooperative mechanism.

When fully aggregated, the dissymmetry factor of the *S*_0_ to *S*_1_ transition (*g*_abs_) is −0.067 (Figure 1e), which is much larger than that of the *S*_0_ to *S*_*n*_ transition (*g*_abs_ = 0.001). Usually, the pure magnetic dipole transitions in the optical frequency range are extremely weak in intensity. Then the associated magnetic transition dipole moment is difficult to determine because extremely small perturbations of the molecular structure (e.g., molecular vibrations) can lead to the mixture of an excited state with an electronic dipole-allowed transition. As a result of this mixing of excited states, the original transition probability, due to the magnetic dipole, can easily be overwhelmed by the admixed electronic dipole strength. However, the significant *g*_abs_ of *S***-H** suggests a magnetically allowed transition between S_1_ and S_0_, offering the possibility to determine the magnetic transition dipole moments.

For determining the magnetic transition dipole moment, we assume *D*_3_ symmetry for the heptazine molecule in the helical aggregate and the lowest excited singlet state of the π–π* nature. The lowest π–π* state must then have *A*_2_ symmetry in order to be connected to the ground state (*S*_0_) via a magnetic dipole-allowed transition. *A*_2_ symmetry for the lowest excited singlet state is consistent with recent quantum chemical studies.^57^ The transition between the S_0_ and S_1_(*A*_2_) excited states is also electric dipole allowed. Both ***m*** and **μ** are parallel to the 3-fold symmetry axis and thus the angle between ***m*** and **μ** can be taken zero. Lastly, when evaluating the magnitude of the component of **μ** parallel to the 3-fold axis, contributions to the total electric dipole in a direction perpendicular to the axis induced via vibronic mixing should be eliminated. This is possible because these in-plane components only contribute to the higher vibronic transitions and a procedure to eliminate these contributions is known.^58^

Therefore, we calculated *m*_1←0,*z*_ through the following functions:^16^
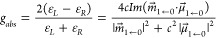
1

Here Δε = ε_*L*_ - ε_*R*_ denotes the circular differential molar decadic extinction coefficient, *c* is the speed of light,  and  are the magnetic and electronic transition dipole moments for the transition from the singlet ground state 0 to the lowest excited singlet state 1. The sign “*Im*” indicates that the imaginary component should be taken. From *S*_0_ to *S*_1_, the electronic dipole moment μ_1←0,*z*_ was determined to be 0.18 D, and the magnetic transition dipole moment *m*_1←0,*z*_ was determined as 0.35 Bohr magneton (see details in SI).

**Figure 2:**
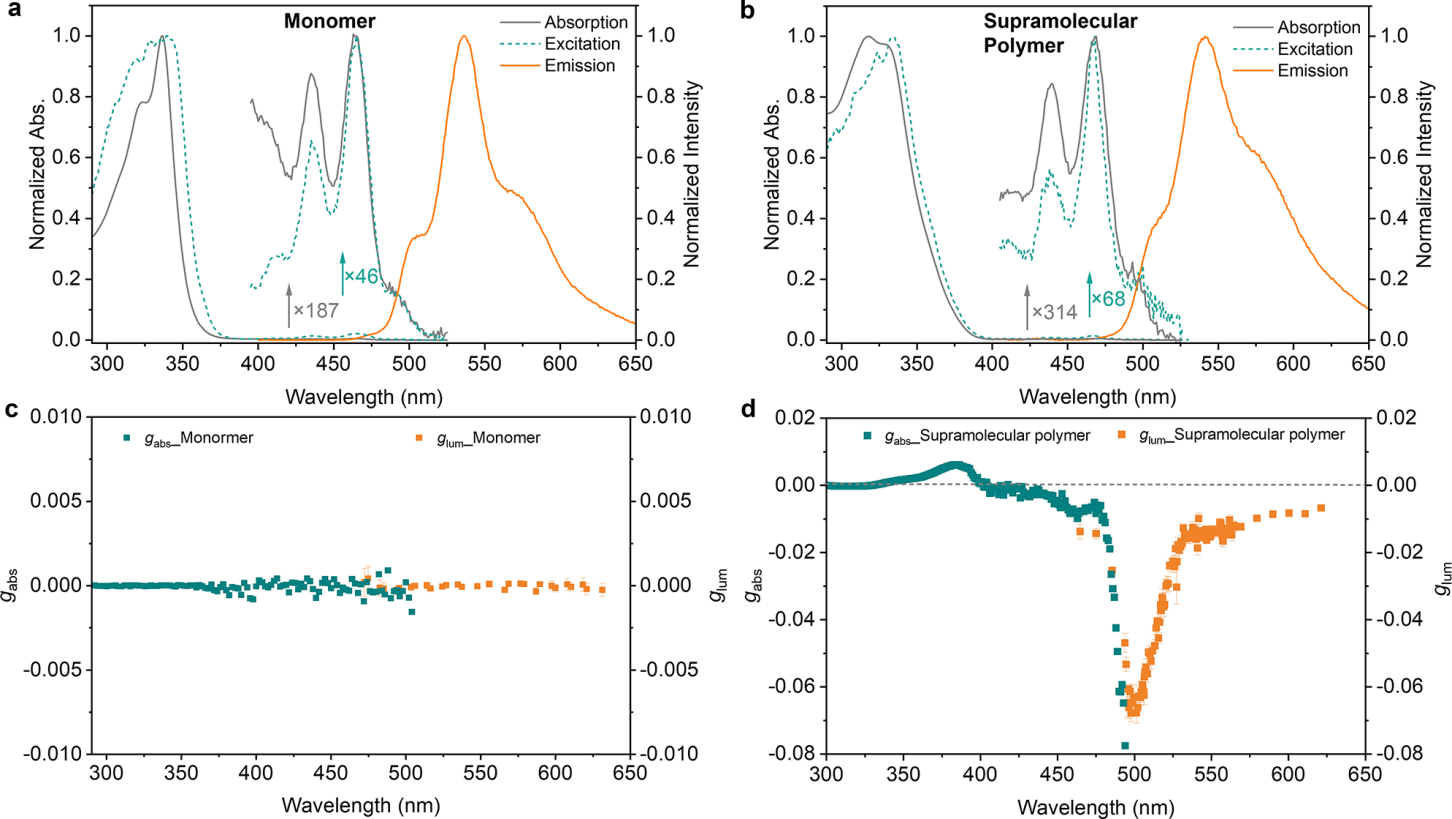
Normalized absorption (gray curve), excitation (green curve), and emission spectra (orange curve) of (a) *S*-**H** monomer (10 μM) in deaerated toluene and (b) supramolecular polymer of *S*-**H** (10 μM) in deaerated toluene/MCH (1/3). *g*_abs_ and *g*_lum_ wavelength curves of the (c) *S***-**H monomer in toluene and (d) the supramolecular polymer of *S*-**H** in deaerated toluene/MCH (1/3).

For the fluorescence associated with the reverse transition from S_1_ to S_0_, we studied the circular polarization, the decay after pulsed excitation, and the quantum yield. The emission and excitation spectra of *S***-H** were measured in deaerated toluene. Monomerically dissolved *S***-H** (10 μM) displayed two excitation bands at 420–515 and 300–375 nm (Figure 2a). Upon excitation, *S***-H** emitted green light, with a maximum at 537 nm in the emission spectra (Figure 2a). Monomeric dissolved *S***-H** did not exhibit circular dichroism (CD) or circularly polarized luminescence (CPL) due to its inherent symmetry of the heptazine core and lack of absorption in the UV–vis region of the chiral alkyl chains. Consequently, both values of *g*_abs_ and *g*_lum_ were found to be zero (Figure 2c). In toluene, a high concentration of *S***-H** is required to obtain a high degree of aggregation. This high concentration of 5–10 mM hindered accurate CPL measurements due to self-absorption and nonpolarized emission contributed from monomers (Figure S6).

**Figure 3:**
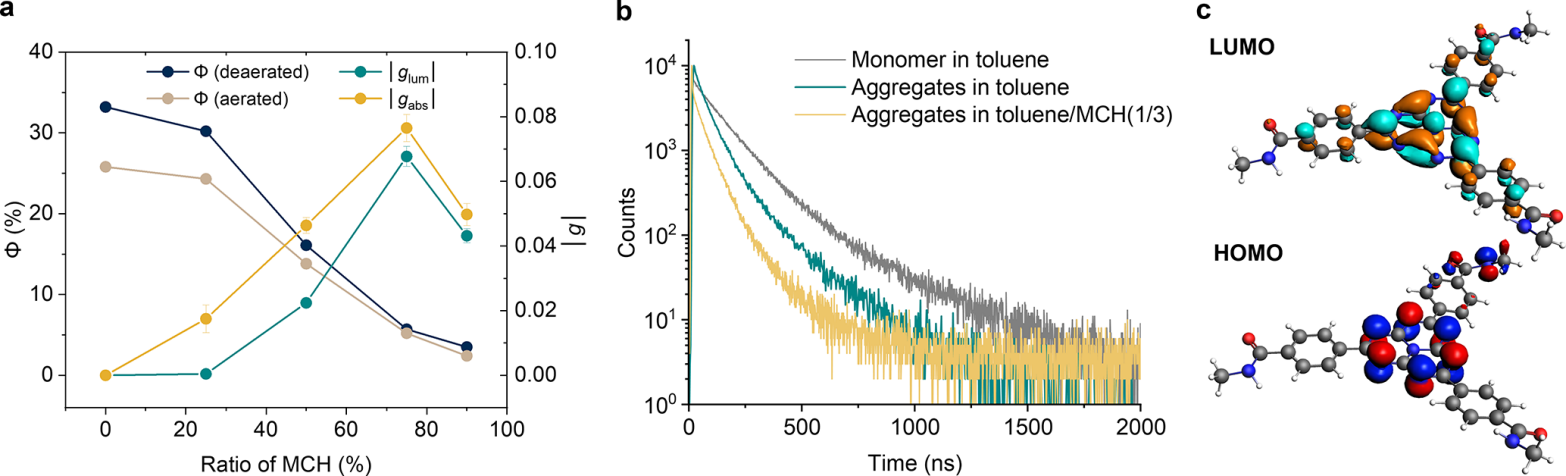
(a) Quantum yield (Φ) and dissymmetric factors (|*g*_lum_|) of *S*-**H** (10 μM) in the toluene/MCH with different solvent ratios. (b) Photoluminescence traces of *S*-**H** monomer (10 μM) in deaerated toluene and supramolecular polymer (10 mM) in deaerated toluene and (10 μM) in deaerated toluene/MCH (1/3). (c) Distribution of electron density in HOMO and LUMO of *S*-**H** calculated at the hybrid-B3LYP level.

To increase the degree of polymerization at lower concentrations, we introduce MCH as a cosolvent. In the mixture of toluene and MCH, UV absorption and CD intensity of *S***-H** (10 μM) at 360 nm exhibit an increasing trend until MCH exceeded 60% (v%) and then leveled off until MCH exceeded 80% (Figure S8), which suggests that ***S*****-H** was fully aggregated in solvent mixtures containing 60%–80% of MCH. Therefore, we used solvent mixtures containing 75% of MCH (toluene/MCH = 1/3) for the following study. The emission spectrum shows a band centered at 537 nm, the same band as that of the monomer (Figure 2b). Unlike the *S***-H** monomer, the supramolecular polymer exhibits intense CPL emission (Figure S7). The maximum values of *g*_abs_ and *g*_lum_ were determined to be −0.078 and −0.068, respectively (Figure 2d). Interestingly, we found that the *g*_lum_ of *S***-H** increased with the ratio of toluene/MCH until 1/3, after which the *g*_lum_ decreased slightly, while the quantum yield (Φ) decreased from 33% to 4% as the MCH ratio increased (Figure 3a). These alterations in photophysics may be attributed to the degree of supramolecular polymerization (Figure S10). Time-correlated single-photon counting studies revealed a multiexponential photoluminescence decay with two distinct emission lifetimes: 54 and 162 ns of supramolecular polymer in the deaerated toluene/MCH with a ratio of 1/3. After obtaining the data, we calculated *m*_1→0,*z*_ through the following functions:
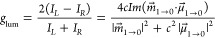
2

Here,  and  correspond to transition dipole moments for the emissive transition from the lowest excited singlet state back to the ground state. The electronic dipole moment μ_1→0,*z*_ was determined to be 0.20 D from the fluorescence decay curve and quantum yield via the Strickler–Berg equation (details in SI). Then, the magnetic transition dipole moment *m*_1→0,*z*_ was determined as 0.36 Bohr magneton, which is close to 0.35 Bohr magneton of *m*_1←0,*z*_. These values are also consistent with the result of 0.4 Bohr magneton from quantum chemical calculation of the magnetic transition dipole moment of the heptazine moiety (C_6_H_3_N_7_ 1,3,4,6,7,9,*9b*-heptaazaphenalene) with *D*_3*h*_ symmetry (see details in SI). These results confirm that the transition between the lowest excited states and the ground state is electronically forbidden but magnetically allowed.

Multiexponential photoluminescence decay was also observed for the monomer of *S***-H** in deaerated toluene with two emission lifetimes of 122 and 351 ns (Figure S13). In the presence of oxygen, the lifetimes of the *S***-H** monomers slightly decrease to 104 and 237 ns, respectively (Figure S13). The emission quantum yield (Φ) decreased from 33% in deaerated toluene to 25% in aerated toluene due to the quenching of triplet excited states by oxygen (Figure 3a). The emission spectrum of *S***-H** remains the same in the presence of oxygen (Figure S12). The small influence of the oxygenation of the sample on the decay of the fluorescence and Φ suggests that the TADF slightly contributes to the emission. Temperature-dependent photoluminescence decay revealed a slight increase of τ upon cooling, suggesting that nonradiative decay is suppressed at low temperatures (Figure S14). In deaerated toluene, the emission lifetimes of supramolecular polymers of *S***-H** were determined to be 62 and 209 ns, whereas in the deaerated toluene/MCH (1/3), the lifetimes were found to be 54 and 162 ns (Figure 3b). Both of them are shorter than the lifetimes of monomers. The reduced lifetime and Φ of supramolecular polymers indicate faster nonradiative decay in the aggregates. In the presence of oxygen, the lifetime and Φ of supramolecular polymers slightly decrease (Figures S15 and S16), which is consistent with the behavior of monomers and suggests the limited contribution from TADF.

To gain further insights into the photophysics of *S***-H**, its electronic structure was calculated using time-dependent density functional theory (TDDFT) with the three-parameter Becke–Lee–Yang–Parr hybrid density functional (hybrid-B3LYP). The highest occupied molecular orbital (HOMO) was found exclusively located on the peripheral nitrogen atoms of the heptazine core, with some on the benzoic amide substituents (Figure 3c). Conversely, the lowest unoccupied molecular orbital (LUMO) is primarily distributed on the carbon atoms and the central nitrogen atom of the core. This negligible overlap between the HOMO and LUMO indicates an intramolecular charge transfer nature of the lowest excited state from nitrogen to carbon, which led to a small electronic transition dipole moment but a large magnetic transition dipole moment between the *S*_1_ and *S*_0_ states. The small electronic transition dipole moment was consistent with the low molar extinction coefficient observed in the UV–vis absorption spectra for the band centered at 465 nm. At the same time, the significant difference in charge distribution on carbon and nitrogen atoms results in a very low exchange energy and an extremely narrow energy gap between the *S*_1_ and *T*_1_ states.

## Conclusion

Incorporating the planar heptazine chromophore into a homochiral supramolecular polymer results in a high degree of circular polarization in the optical transition between the ground and the lowest excited singlet state. From the circular polarization, the magnetic transition dipole moment for this forbidden transition can be determined and is found to be a 0.4 Bohr magneton. The high value of the magnetic transition dipole moment is consistent with the limited overlap of HOMO and LUMO orbitals and the intramolecular charge transfer nature of the lowest excited state from nitrogen to carbon. This spatial separation of the orbitals is responsible for the small singlet–triplet energy splitting and the charge transfer nature. The present study offers a method for probing the lowest excited states of conjugated flat chromophores with a magnetically allowed transition via supramolecular polymerization. We propose that such a photophysical understanding will pave the way for designing heptazine-based chiral materials for spin-controlled reactions such as water splitting.

## Supporting Information Available

The synthesis of *S***-H**, NMR spectra for all compounds, FT-IR spectra, AFM images, additional experimental details, materials, and methods, including modeling and calculations, are shown in the Supporting Information (PDF)
