# Supplementary material for: Supramolecular Polymerization as a Tool to Reveal the Magnetic Transition Dipole Moment of Heptazines
Source: J Am Chem Soc. 2024 May 30;146(23):15843–9. doi: 10.1021/jacs.4c02174 (PMC11177250; doi:10.1021/jacs.4c02174)
Supplement: Supplementary file 1 — ja4c02174_si_001.pdf [file ja4c02174_si_001.pdf]

Supramolecular Polymerization as a Tool to Reveal the Magnetic Transition Dipole Moment of Heptazines

Fan Xu, Hao Su, Joost J. B. van der Tol, Stef A. H. Jansen, Youxin Fu, Giulia Lavarda, Ghislaine Vantomme, Stefan Meskers,* and E. W. Meijer*

Table of Contents

1. Materials. 2
2. General. 2
3. Synthesis. 2
4. AFM measurements. 3
5. UV-vis and CD spectroscopy 4
6. Computational analysis of CD data. 5
7. FT-IR measurements. 7
8. VT-NMR measurements. 7
9. Circularly polarized luminescence measurements. 7
10. UV-vis and CD spectroscopy in the mixture of toluene and MCH. 8
11. Photoluminescence spectra. 10
12. Photoluminescence decay. 10
13. Determination of the electric and magnetic transition dipole moment of the $S_1 \leftarrow S_0$ absorption band of heptazine. 12
14. Determination of the electric and magnetic transition dipole moment of the $S_1 \rightarrow S_0$ luminescence band of heptazine. 15
15. Calculation of the magnetic transition dipole moment for the heptazine core molecule..17
16. DFT calculation. 19
17. IR and NMR data. 19
References: 26

1. Materials.

All commercially available chemicals were purchased from Acros, Aldrich, or TCI, and were used as received. Solvents used in the reactions were dried using an MBraun SPS-800 solvent purification system or purchased from Acros and Aldrich. The water (ULC/MS grade) used in sample preparation was purchased from Biosolve.

2. General.

Column chromatography was performed using 200-425 mesh silica gel. NMR spectra were recorded at 25 °C on a Bruker ASCEND 400MHz (^{1}H: 400 MHz, ^{13}C: 101 MHz) and Varian Unity Plus (^{1}H: 500 MHz, ^{13}C: 125 MHz) spectrometers. Chemical shifts (δ) are expressed relative to the resonances of the residual non-deuterated solvent for ^{1}H NMR [$CDCl_3$: ^{1}H(δ) = 7.26 ppm, C_6D_6: 7.16 ppm, DMF-d$_7$: 8.03 ppm, THF-d$_8$: 3.58 ppm] and ^{13}C NMR [$CDCl_3$: ^{13}C(δ) = 77.2 ppm, C_6D_6: 128.06 ppm, DMF-d_7: 163.15 ppm, THF-d_8: 67.21 ppm]. Absolute values of the coupling constants are given in Hertz (Hz), regardless of their sign. Multiplicities are abbreviated as singlet (s), doublet (d), doublet of doublets (dd), triplet (t), triplet of doublets (td), quartet (q), multiplet (m), and broad (br). Infrared spectra (IR) of solid samples were measured with a PerkinElmer Spectrum Two spectrometer and the solution sample was recorded on a Shimadzu IR-Tracer 100 Fourier-Transform Infrared spectrometer equipped with a liquid cell with windows of calcium fluoride. MALDI-TOF-MS spectra were recorded with Bruker Autoflex Speed. The fluorescence quantum yields (Φ) measurement was performed in an Edinburgh instruments FS5 spectrofluorometer by using an integrating sphere mounted in the sample chamber.

3. Synthesis.

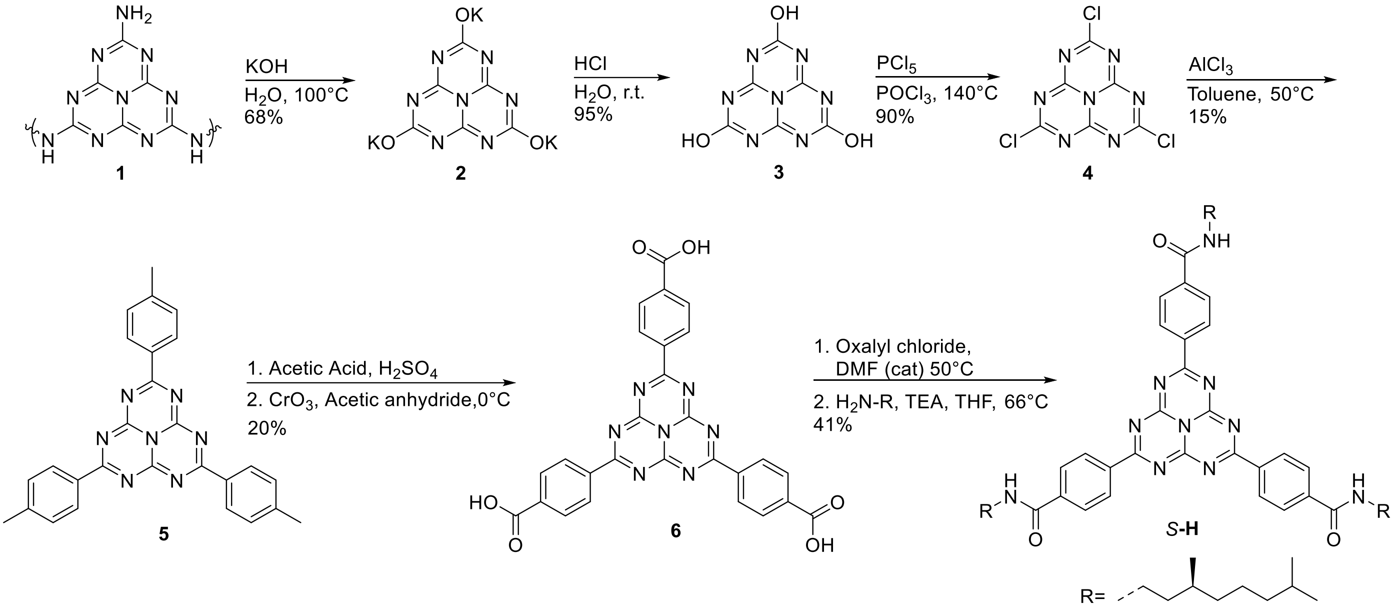

Scheme S1. Synthesis of *S*-**H**.

Potassium Cyamelurate (2)

Melon pellets were pulverized with a pestle of a mortar. A round bottom flask (250 ml) was charged with 10 g of the yellow powder (45 mmol, 1 eq.) and 90 ml of 3 M KOH (275 mmol, 6 eq.). Subsequently, the reaction was refluxed for 3 h. After the reflux, the mixture was quickly filtrated in a warmed Büchner funnel. Grey impurities separated and a white crystal formed in the filtrate upon cooling down. The filtrate was filtrated. The second residue was washed with ethanol thrice. Afterwards, the residue was dried in a vacuum and collected. A white powder was obtained. Yield: 68%. IR (cm^{-1}): 3064, 1644, 1515, 1471, 1404, 1151, 813.

Cyameluric Acid (3)

A round bottom (500 ml) was charged with 10.8 g potassium cyamelurate (5.96 mmol, 1 eq.) and 50 ml of demi water. Subsequently, 130 ml of 1 M HCl (130 mmol, 4 eq.) was added dropwise. A white solid precipitate was observed. After the mixture had reacted for 10 minutes, it was filtrated. The filtrate was washed thrice with water and dried in a vacuum. A white powder was obtained. Yield: 95%. IR (cm^{-1}): 2985, 1610, 1474, 1399, 1310, 1168, 952, 826, 786, 628, 537. MALDI-TOF-MS: m/z calculated: 221.03, found: 221.14.

Cyameluric Chloride (4)

The reaction was carried out under inert conditions and dried glassware. A round Schleck bottom flask (250 ml) was charged with cyameluric acid (2 g, 9.04 mmol, 1 eq.) and 50 ml $POCl_3$. PCl_5 (8.5 g 40.7 mmol, 4.5 eq.) was added slowly to the mixture. The mixture was refluxed for 4 h. The generation of an acidic gas was observed, and it was neutralized by passing it through an aqueous

NaOH 1 M solution. After 2 hours of refluxing the color had changed from white to yellow. In addition, the mixture was less turbid than in the beginning. After the reflux, the $POCl_3$ was removed via distillation. The temperature was raised to 140 °C and the mixture was exposed to an argon flow, resulting in the complete removal of the $POCl_3$ and sublimating the PCl_5 excess to the upper part of the vessel. The product was obtained as a yellow powder. Yield: 90%. ^{13}C NMR (100 MHz, THF-d_8): δ = 175.4, 158.6. IR (cm^{-1}): 1601, 1498, 1302, 1200, 1088, 970, 824, 648, 579.

*2,5,8-tri-p-tolyl-1,3,3a1,4,6,7,9-Heptaazaphenalene (**5**)*

A 100 ml round bottom flask was charged with 40 ml toluene (376 mmol, 40 eq.) and 2.4 grams of anhydrous $AlCl_3$ (14.47 mmol, 2 eq.). The suspension was heated to 60 °C. 2.5 g of cyameluric chloride (9.04 mmol, 1 eq.) was added slowly. Upon the addition of cyameluric chloride, the color changed to dark red. After the addition of cyameluric chloride, the reaction was cooled down to RT and left to react for 14 h. 100 ml water was added, and the mixture turned yellow. The solids were collected by filtration and recrystallized from a large amount of DMF. The product was obtained as a yellow solid. Yield: 15%. ^{1}H NMR (400 MHz, C_6D_6): δ =2.02 (s, 3H), 7.04 (d 2H) 8.80 (d, 2H). ^{13}C NMR (100MHz, C_6D_6): δ= 180.62, 168.01, 145.18, 133.11, 130.66, 129.84, 21.57. IR (cm^{-1}): 3033, 1621, 1587,1509, 1492, 1405, 1384, 1296, 1223, 1174, 1037, 1019, 929, 851, 815, 796, 738, 684, 609, 516, 477. MALDI-TOF-MS: m/z calculated 443.19, found: 444.20.

*4,4',4''-(1,3,3a1,4,6,7,9-*H*eptaazaphenalene-2,5,8-triyl)tribenzoic acid (**6**)*

A 100ml round bottom flask was charged with 1.21 g of 2,5,8-tri-p-tolyl-1,3,3a1,4,6,7,9-Heptaazaphenalene (2.73 mmol, 1 eq), 36.20 g of acetic acid (602.81 mmol, 220 eq.) and 4.02 g of sulphuric acid (41.02 mmol, 15 eq.) at 0°C. This mixture was allowed to be stirred for 10 minutes. 4.92 g of CrO_3 (49.23 mmol, 18 eq.) was added slowly, followed by 2.2 g of acetic anhydride (21.88 mmol, 8 eq.). Upon the addition of the CrO_3, the mixture turned green. The reaction mixture was allowed to be stirred overnight at room temperature. The reaction mixture was poured into 300 ml of distilled water. A yellow solid was collected via filtration and dissolved in 100 ml 6 M NaOH. The resulting mixture was acidified with 30 ml concentrated HCl, resulting in a yellow precipitation. The yellow precipitation was collected via filtration. The filtrate was recrystallized from hot DMF. The yellow crystals were collected via filtration and washed with 100 ml 1 M HCl. The yellow powder was dried in a vacuum. The product was obtained as a yellow powder. Yield: 20%. ^{1}H NMR (400 MHz, DMF-d_7): δ = 8.27 (d, 2H), 8.62 (d, 2H), 13.79 (s, 1H,). ^{13}C NMR (100MHz, DMF-d_7): δ = 174.6, 167.0, 160.5, 138.4, 136.2, 130.15, 129.9. IR (cm^{-1}): 3056, 1715, 1589, 1498, 1408, 1373, 1303, 1220, 1105, 1014, 924, 875, 830, 789, 718. MALDI-TOF-MS: m/z calculated: 533.11, found: 533.19.

*4,4',4''-(1,3,3a1,4,6,7,9-*H*eptaazaphenalene-2,5,8-triyl)tris(N-octylbenzamide) (S-**H**)*

The reaction was carried out under inert conditions in dried glassware under argon. 100 mg 4,4',4''-(1,3,3a1,4,6,7,9-Heptaazaphenalene-2,5,8-triyl)tribenzoic acid (0.19 mmol, 1 eq.) were added to 10 mL oxalyl chloride. One drop of DMF was added as the catalyst. The mixture was stirred for 2 h at 50 °C. Oxalyl chloride was removed by evaporation. The generated acid chloride was redispersed in 10 mL dry THF and added to a 10 mL THF solution of (*S*)-3,7-dimethyloctan-1-amine (0.66 mmol, 3.5 eq.) and triethylamine (0.66 mmol, 3.5 eq.). The mixture was stirred at 66 °C for one hour. The reaction mixture was cooled down and concentrated. The concentrate was columned using eluents $CHCl_3$/MeOH (95/5%). After the column, the solvent was removed, and the powder was precipitated in methanol to obtain a yellow-orange powder. Yield: 41%. ^{1}H NMR (400MHz, $CDCl_3$): δ = 0.89 (d, 18H), 0.98 (9H, d), 1.13-1.40 (21H, m), 1.45-1.55 (6H, m), 1.64-1.74 (3H, m), 6.57 (s, 3H), 7.62 (6H, d), 8.34 (6H, d). ^{13}C NMR (100MHz, $CDCl_3$): δ = 174.54, 167.97, 157.97, 140.30, 134.72, 129.95, 126.89, 39.31, 38.61, 37.32, 36.57, 31.11, 28.01, 24.79, 22.77, 22.65, 19.59. IR (cm^{-1}): 3303, 2953, 2925, 2868, 1642, 1618, 1593, 1535, 1500, 1460, 1407, 1385, 1302, 1223, 1170, 1158, 1110, 1017, 923, 870, 828, 720, 622. MALDI-TOF-MS: m/z calculated: 950.63, found: 950.73.

4. AFM measurements.

Samples were prepared by spin-coating 20 µL of 160 µM toluene solution of *S*-**H** onto freshly cleaved mica followed by overnight drying under ambient condition. A spin-coating speed of 1000 rpm was used. All measurements were performed on a Cypher Environmental Scanner (ES) equipped with a closed cell and a heater-cooler stage. Topological measurements were performed using a normal laser diode and recorded using AC160TS probes and a scan rate of 3 Hz. The contrast of topological images was further enhanced using first-order plane fit and flattening using Gwyddion v2.60.

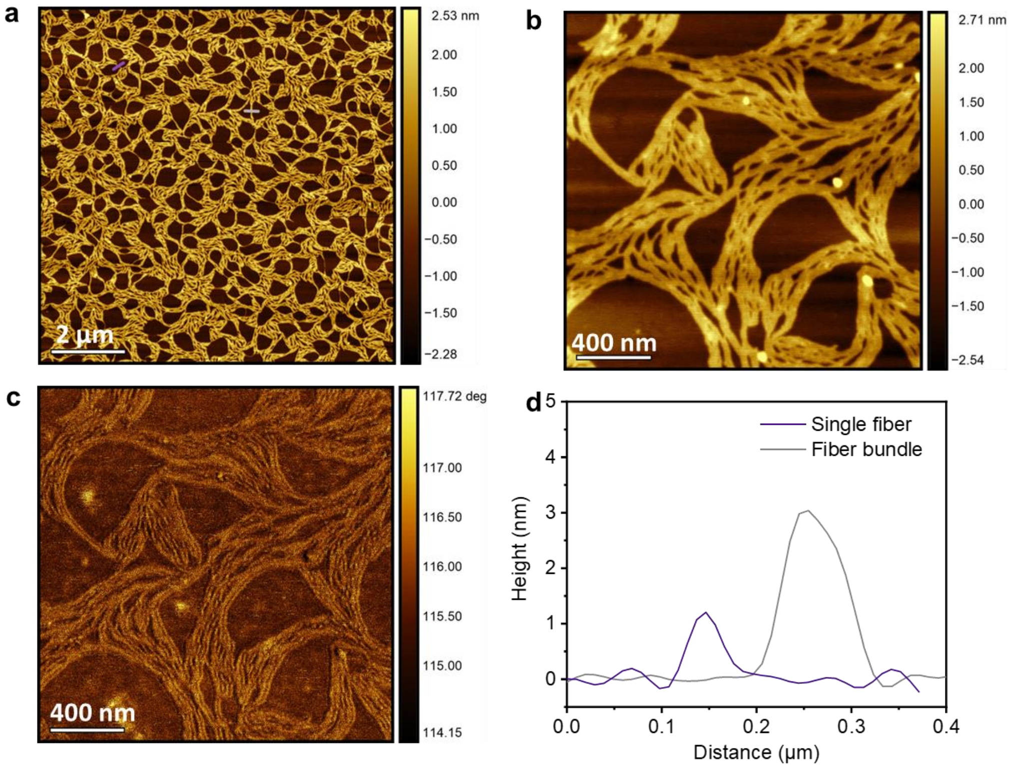

Figure S1. AFM height images of *S*-**H** supramolecular polymer prepared from a toluene solution (160 μM) measured in an area of (a) 10×10 μm (b) 2×2 μm. (c) AFM phase image of *S*-**H** supramolecular polymer corresponding to Figure S1b. (d) Height profile of a single fiber (purple) and a fiber bundle (gray), as indicated in Figure S1a, suggests a single fiber diameter of 1.2 nm. The discrepancy between the height and width of the measured feature originates from the conventionally observed AFM tip-sample convolution in the x-y plane.

5. UV-vis and CD spectroscopy.

Stock solution (1 mM) was prepared by weighing the compound into a screw-capped vial and adding the required amount of $CHCl_3$. The stock solutions were sonicated for 30 seconds. The sample solutions were prepared via aliquoting a certain volume of $CHCl_3$ stock solution into a sealable vial. The $CHCl_3$ was removed by N_2-blow-drying for 1 h followed by overnight drying under ambient conditions. Then proper volume of solvent was added to the vial to give the final concentration. The sample was then sonicated for about 30 s and heated to be fully dissolved. Samples were then transferred into quartz cuvettes with a path length of 1 or 10 mm, depending on each sample. CD spectra were recorded on the Jasco J-815 Circular Dichroism Spectrometer. UV-vis spectra were recorded on a Cary 3500 spectrometer and Jasco J-815 Circular Dichroism Spectrometer.

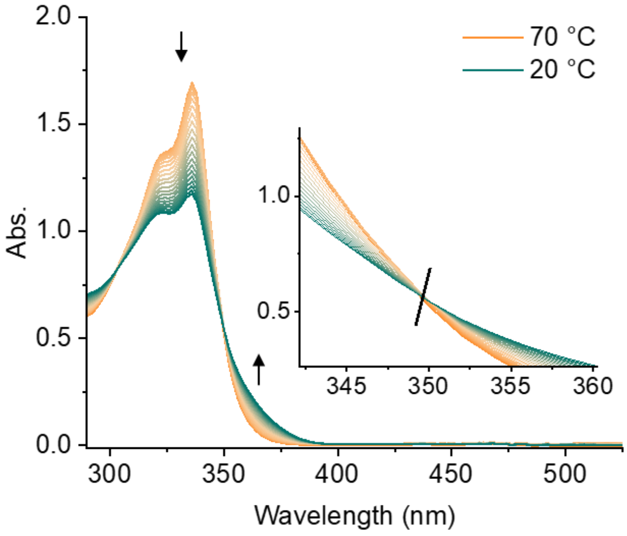

Figure S2. UV-vis absorption spectra of a toluene solution of *S*-**H** (160 μM) upon cooling with a rate of –2 K/min.

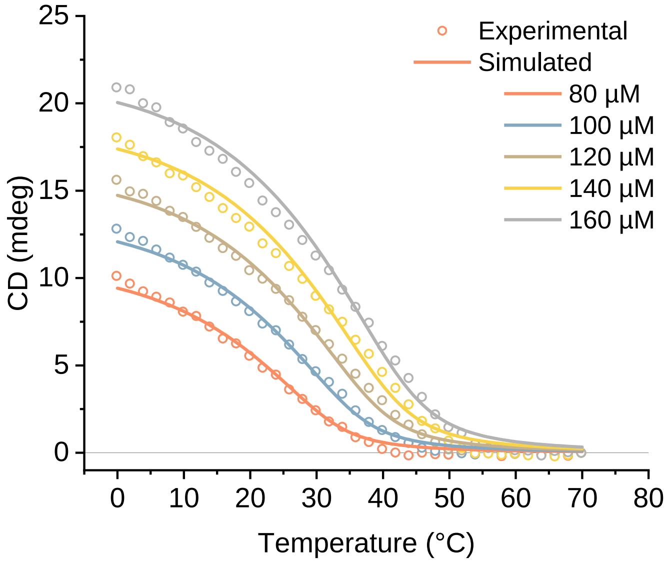

Figure S3. Cooling curves of *S*-**H** in toluene at different concentrations fitting with the thermodynamic mass-balance model.

6. Computational analysis of CD data.

The supramolecular polymerization is modeled using thermodynamic mass-balance expressions.[1] In the model, the polymers (P) are assumed to grow through monomer (M) addition and dissociation at the chain ends. The reactions that describe the cooperative pathway, for which a nucleus size of 2 is assumed, are:

$$\begin{aligned}
&\mathrm{M}+\mathrm{M} \underset{}{\overset{K_n}{\rightleftharpoons}} \mathrm{P}_2 \\
&\mathrm{P}_2+\mathrm{M} \underset{}{\overset{K_e}{\rightleftharpoons}} \mathrm{P}_3 \\
&\mathrm{P}_3+\mathrm{M} \underset{}{\overset{K_e}{\rightleftharpoons}} \mathrm{P}_4 \\
&\quad\vdots \\
&\mathrm{P}_j+\mathrm{M} \underset{}{\overset{K_e}{\rightleftharpoons}} \mathrm{P}_{j+1} \\
&\quad\vdots
\end{aligned} \tag{E1}$$

with K_n the nucleation constant and K_e the elongation constant of the nucleated pathway. Assuming the activity of the chemical species is equal to their concentrations, the concentration of monomers in i-mer in the cooperative aggregates in thermodynamic equilibrium can then be expressed as a function of the free monomer concentration with:

$$[\mathrm{P}_i] = i \cdot \sigma \cdot K_\mathrm{e}^{i-1} \cdot [\mathrm{M}]^i \text{ for } i \geq 2 \quad \text{(E2)}$$

where [M] is the equilibrium monomer concentration and σ is the cooperativity parameter, which is $\sigma = K_n/K_e$.

The total concentration of M in the system is the sum of monomers and nucleated aggregates:

$$\begin{aligned} [\mathrm{M}]_\mathrm{tot} &= [\mathrm{P}]_\mathrm{tot} + [\mathrm{M}] \\ &= \left(\sum_{i=2}^{\infty} [\mathrm{P}_i] \right) + [\mathrm{M}] \\ &= \left(\sum_{i=1}^{\infty} i \cdot \sigma \cdot K_\mathrm{e}^{i-1} [\mathrm{M}]^i \right) - \sigma \cdot [\mathrm{M}] + [\mathrm{M}] \end{aligned} \quad \text{(E3)}$$

With standard expressions for converging series, the summation in equation (E3) can be solved and the mass-balance equation for the system can be obtained:

$$[\mathrm{M}]_\mathrm{tot} = (1-\sigma) \cdot [\mathrm{M}] + \frac{\sigma \cdot [\mathrm{M}]}{(1 - K_\mathrm{e} \cdot [\mathrm{M}])^2} \quad \text{(E4)}$$

This equation is solved in Matlab®, using a custom written binary search algorithm, to obtain the free monomer concentration. The free monomer concentration is then used to calculate the concentration of nucleated aggregates.

The binding constant K_e is rendered temperature-dependent through the van 't Hoff expression:

$$K_\mathrm{e} = \exp\left(\frac{-\Delta G_\mathrm{e}}{R \cdot T} \right) = \exp\left(\frac{-\Delta H_\mathrm{e}}{R \cdot T} + \frac{\Delta S}{R} \right) \quad \text{(E6)}$$

With R the gas constant, T the temperature, ΔH_e and ΔS the enthalpy and entropy of elongation, respectively.

The nucleation penalty NP is related to the cooperativity parameter σ via:

$$\sigma = e^{\frac{-NP}{R \cdot T}} \quad \text{(E6)}$$

The above-described model is fitted to the CD signal at 360 nm. To predict the spectroscopic response, the concentration of every aggregate type (M and P) is multiplied by the molar absorbance or molar ellipticity for the specific aggregate types:

$$\theta = \theta_\mathrm{M} \cdot [\mathrm{M}] + \theta_\mathrm{P} \cdot [\mathrm{P}]_\mathrm{tot} \quad \text{(E7)}$$

where θ is the ellipticity in mdeg, θ_i is the molar ellipticity of species i in mdeg·M·cm^{-1}. The molar ellipticity of the monomers θ_M is fixed at 0. The fit parameters were ΔH_e, ΔS, NP and θ_P.

The differences between the simulated data and the experimental data were combined in a cost vector. The minimization of the cost vector was performed using the Matlab® lsqnonlin function with the Levenberg-Marquardt algorithm to obtain optimal values for the thermodynamic parameters of the supramolecular polymerization. To ensure that the solution is at the global minimum, the fits were performed with a minimum of 500 initial parameter sets. The initial parameter sets were defined using a Latin Hypercube Sampling method, implemented with the Matlab® function lhsdesign. To ensure reasonable values of the set of starting parameters in the fitting procedure, ΔG_e was sampled between –60 and –30 kJ/mol, ΔS between –200 and –50 J/mol·K, ΔG_n between –50 and –20 kJ/mol and θ_P between 1.0×10^6 and 1.2×10^6 mdeg·M·cm^{-1}. The final fitting parameters that resulted in the lowest norm of the residual cost vector were selected as the best fit.

Table S1. Thermodynamic parameters for supramolecular polymerization of *S*-**H** were obtained from fitting mass balance model to CD cooling curves.

ΔH_e (kJ/mol)	ΔS (J/(mol*K))	NP (kJ/mol)
-49.2	-84.0	10.2

7. FT-IR measurements.

Toluene solution of *S*-**H** (200 μM) was placed in a cell with windows made of CaF_2.

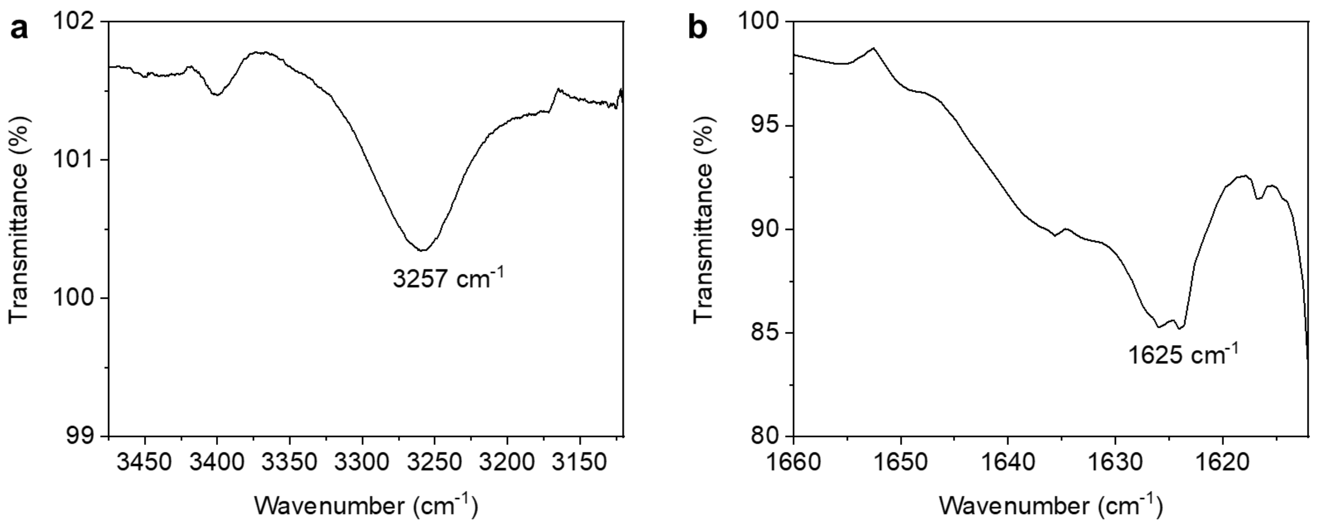

Figure S4. FT-IR spectra of *S*-**H** (200 μM) in toluene.

8. VT-NMR measurements.

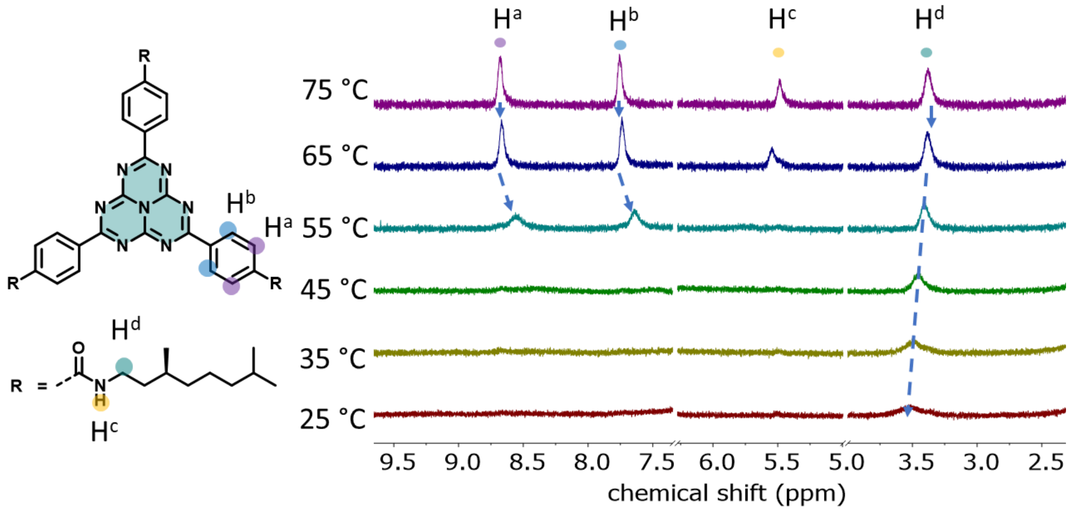

Figure S5. VT-^{1}HNMR spectra of *S*-**H** (200 μM) in toluene-d_8.

9. Circularly polarized luminescence measurements.

Circularly polarized luminescence measurements were performed using a homemade CPL/LPL spectrometer, constructed by photoelastic modulation at 50 kHz, parallel multichannel detection, single photon counting electronics and an Hg lamp. Samples

at concentrations lower than 0.2 mM are excited at 313 nm, while samples at a higher concentration are excited at 405 nm.

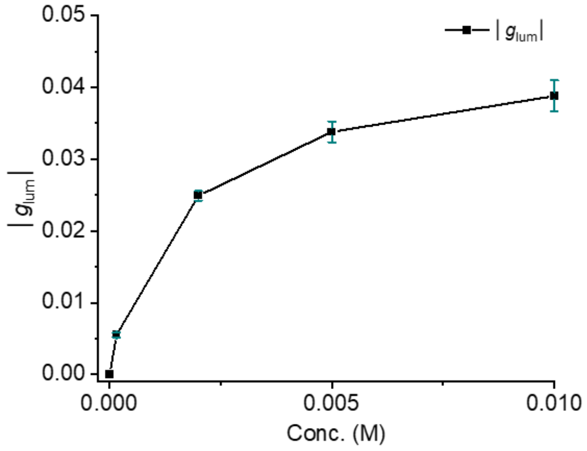

Figure S6. The value of dissymmetry factor of luminescence (| g_{lum} |) at 493 nm of *S*-**H** in toluene at different concentrations.

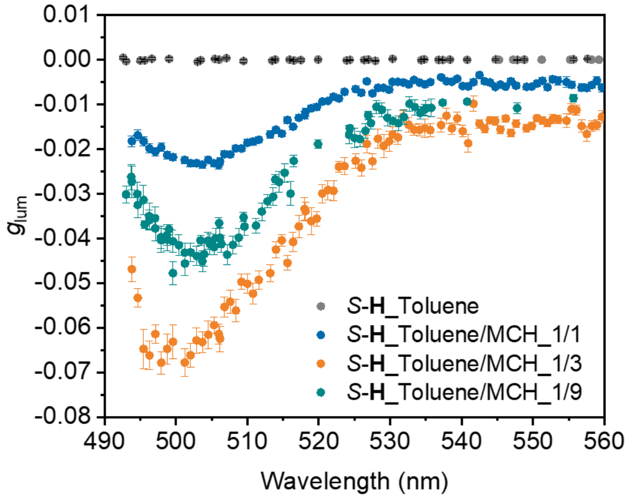

Figure S7. Dissymmetry factor g_{lum} as a function of wavelengths for *S*-**H** supramolecular polymers in toluene (black curve) and the mixture of toluene and MCH with a ratio of 1/1 (blue curve), 1/3 (orange curve), and 1/9 (green curve).

10. UV-vis and CD spectroscopy in the mixture of toluene and MCH.

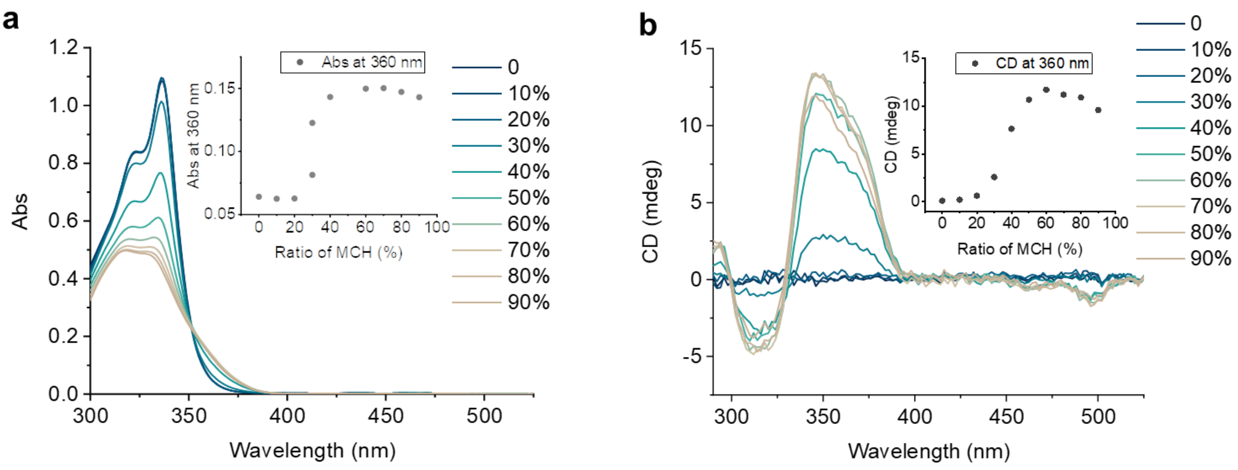

Figure S8. (a) UV-vis absorption (b) CD spectra of *S*-**H** in the solvent mixture of toluene and MCH with different ratios. Inset: absorption and CD signal at 360 nm of *S*-**H** in the mixture of toluene and MCH at various ratios.

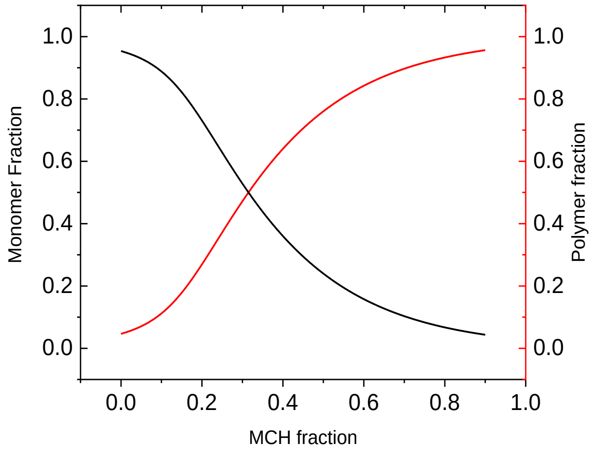

Figure S9. Calculated monomer and supramolecular polymer fractions in the solvent mixture at different ratios. The calculations were performed with the optimized thermodynamic parameters for *S*-**H** in toluene with an additional dependency of the ΔG_e to the MCH fraction ($m_{MCH} = -10.8$ kJ/mol).[2]

$$K_e = exp\left(\frac{-\Delta G_e}{R \cdot T}\right) = exp\left(\frac{-\Delta G_e^{\circ} + m_{MCH} \cdot f_{MCH}}{R \cdot T}\right) \quad \text{(E8)}$$

with R the gas constant, T the temperature, ΔG_e° the Gibbs free energy of elongation of the cooperative polymerization, ΔG_e the cosolvent-corrected Gibbs free energy of elongation of the cooperative polymerization, and m_{MCH} the solvent dependency parameter of the elongation process to MCH, which is present in solvent fraction f_{MCH}.

To eliminate the number of monomers in the supramolecular, we calculate the number-average degree (DPn) of polymerization at room temperature using the optimized parameters obtained from fitting the solvent titration curve (Figure S8).[1] As shown in Figure S10, the calculation suggests that DPn must be about 9 to achieve the highest dissymmetry factor.

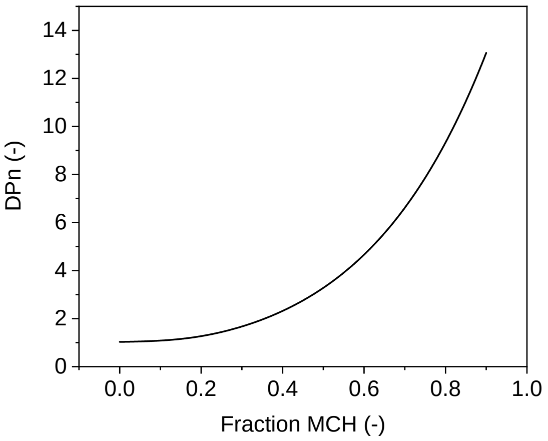

Figure S10. Calculated number-average degree of polymerization in the solvent mixture of MCH and toluene at different ratios.

We realized that we could increase the polymer fraction of *S*-**H** by introducing MCH as a bad solvent in the samples. The addition of MCH increased the degree of aggregation, as indicated by the UV/Vis and CD spectra. At high fractions of MCH (> 0.8), we observed an unexpected decrease in the degree of aggregation, which is likely caused by the formation of different aggregates or precipitation of *S*-**H**. The formation of different aggregates was confirmed by AFM images of *S*-**H** supramolecular polymers in MCH/Toluene (9/1) (Figure S10). The assembled *S*-**H** shows a pancake shape with a height of 15 nm and a diameter of about hundreds of nm, in which the fiber bundles are likely intertwined. The formation of bundles by increasing the ratio of bad solvents was also observed in our group's previous paper. Thus, it is important to keep in mind that under the condition of a higher ratio of MCH, *S*-**H** might not only be in one-dimensional cooperative polymers, and proper MCH fractions should be used if it is desired to have only one-dimensional supramolecular polymers of *S*-**H**.

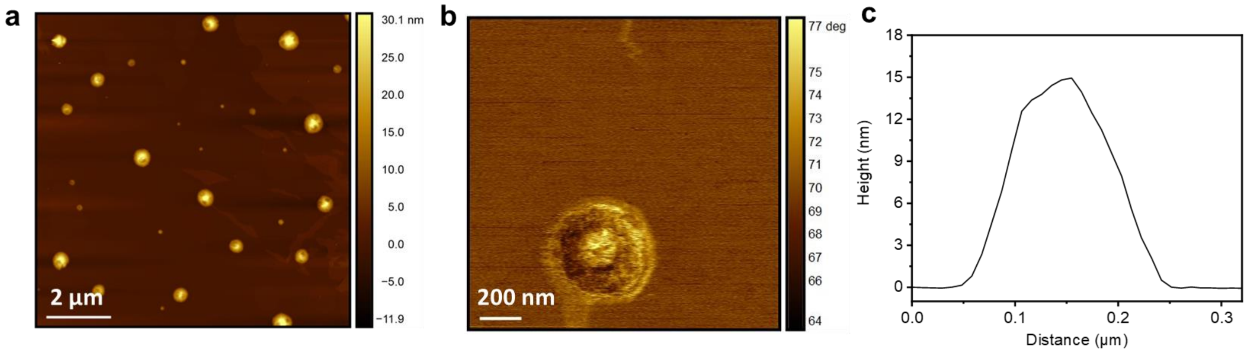

Figure S11. AFM height images of *S*-**H** supramolecular polymer prepared from toluene/MCH (1/9) (10 µM) measured in an area of (a) 10×10 µm (b) AFM phase image of *S*-**H** supramolecular polymer corresponding measured in an area of 2×2 µm (d) Height profile of a plate.

11. Photoluminescence spectra.

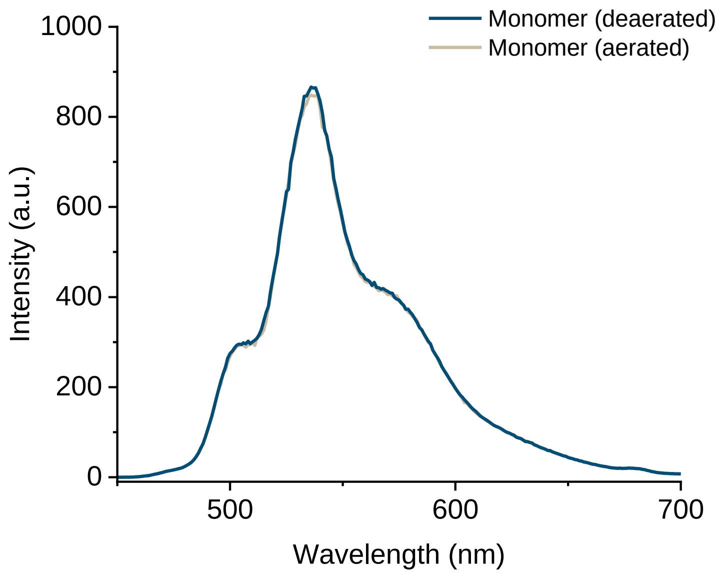

Figure S12. Emission spectra of *S*-**H** monomer in deaerated and aerated toluene.

12. Photoluminescence decay.

Time-correlated single photon counting was measured using an Edinburgh Instruments LifeSpec-PS spectrophotometer with 400 nm pulsed diode laser (LDH-C 400 driven by a PDL-800B) excitation.

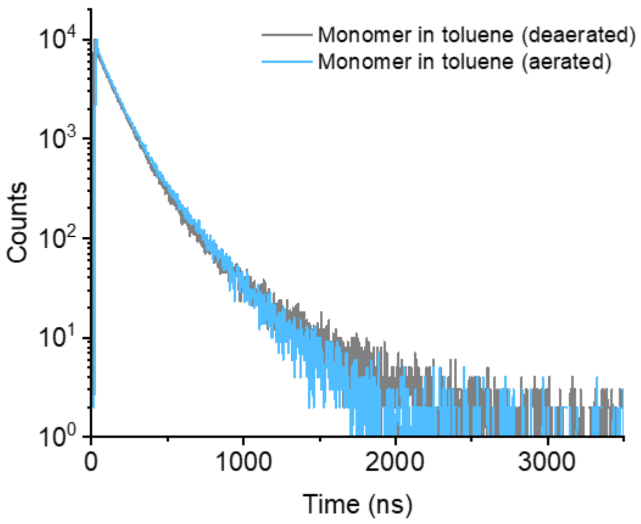

Figure S13. Photoluminescence traces of *S*-**H** monomer (10 µM) in deaerated and aerated toluene, separately.

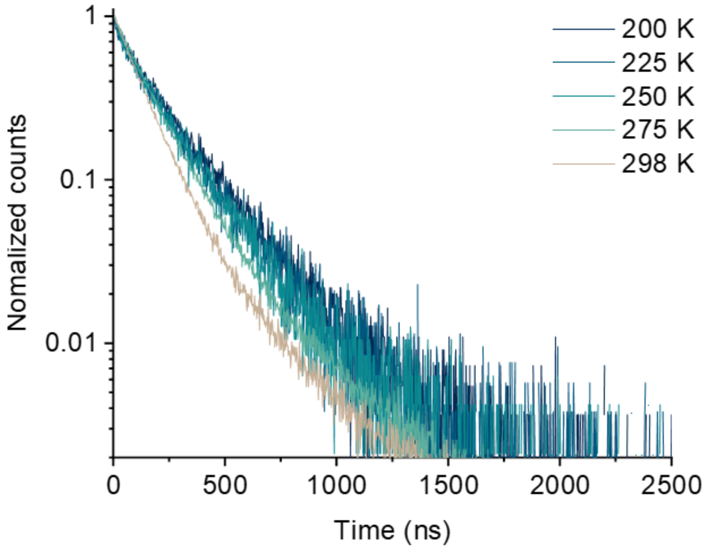

Figure S14. Normalized temperature-dependent photoluminescence traces of *S*-**H** (10 µM) in deaerated toluene.

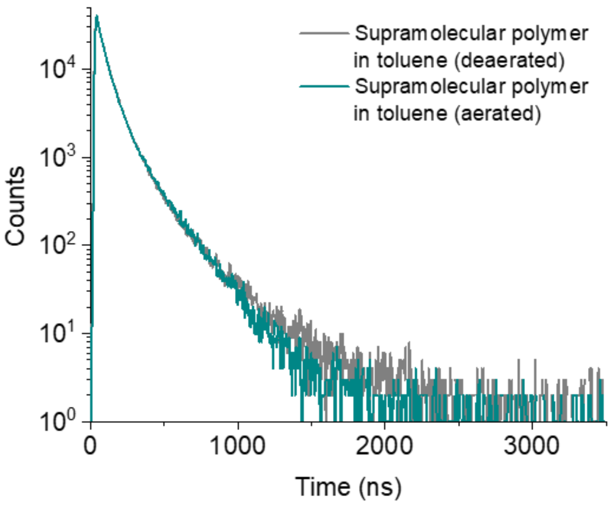

Figure S15. Photoluminescence traces of ***S*-H** supramolecular polymer (10 mM) in deaerated and aerated toluene, separately.

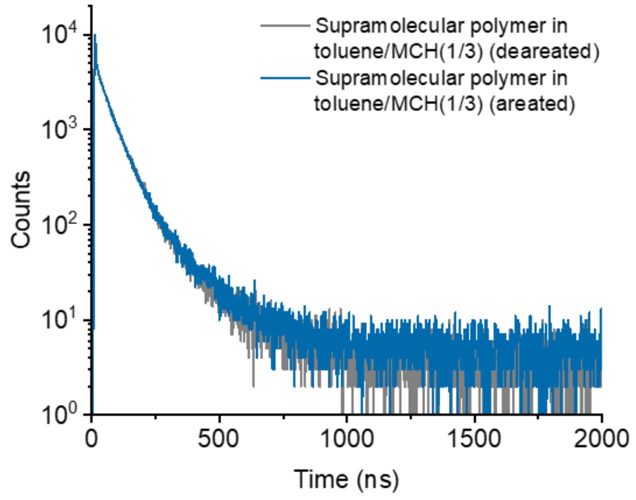

Figure S16. Photoluminescence traces of supramolecular polymer (10 µM) in deaerated and aerated mixture of toluene and MCH (1/3).

13. Determination of the electric and magnetic transition dipole moment of the $S_1 \leftarrow S_0$ absorption band of heptazine.

The lowest excited state of the core heptazine moiety ($C_6H_3N_7$ 1,3,4,6,7,9,9b-Heptaazaphenalene) with D_{3h} symmetry has been studied extensively through quantum chemical calculations.[3,4,5] The lowest excited singlet state is of ππ* orbital nature and A'_2 symmetry. The state can be accurately described in terms of a single electron excitation from the A''_1 HOMO to the A''_2 LUMO orbital. The transition from the ground state to this lowest excited singlet state in electric dipole is forbidden but magnetic dipole is allowed in the z direction perpendicular to the aromatic plane.

Pure magnetic dipole transitions in the optical frequency range are extremely weak in intensity.[6] The associated magnetic transition dipole moment is difficult to determine because extremely small perturbations of the molecular structure e.g. molecular vibrations can lead to the admixture of an excited state with an electric dipole-allowed transition. As a result of this mixing of excited states, the original transition probability due to the magnetic dipole can easily be overwhelmed by the admixed electric dipole strength.

Chiral molecules offer a unique opportunity to determine the magnetic m and electric μ transition dipole moments through measurements of the circular polarization in absorption and emission. The degree of circular polarization in absorption (g_{abs}) and luminescence (g_{lum}) depends on the relative magnitude and orientation of the magnetic and electric transition dipole moments:

$$g_{abs} = \frac{2(\varepsilon_L - \varepsilon_R)}{\varepsilon_L + \varepsilon_R} = \frac{4c Im(\vec{m}_{1\leftarrow 0} \cdot \vec{\mu}_{1\leftarrow 0})}{|\vec{m}_{1\leftarrow 0}|^2 + c^2 |\vec{\mu}_{1\leftarrow 0}|^2} \quad \text{(S1)}$$

$$g_{lum} = \frac{2(I_L - I_R)}{I_L + I_R} = \frac{4c Im(\vec{m}_{1\rightarrow 0} \cdot \vec{\mu}_{1\rightarrow 0})}{|\vec{m}_{1\rightarrow 0}|^2 + c^2 |\vec{\mu}_{1\rightarrow 0}|^2} \quad \text{(S2)}$$

Here $\Delta\varepsilon = \varepsilon_L - \varepsilon_R$ denotes the circular differential molar decadic extinction coefficient, c the speed of light, $\vec{m}_{1\leftarrow 0}$ and $\vec{\mu}_{1\leftarrow 0}$ the magnetic and electric transition dipole moments for the transition from the singlet ground state 0 to the lowest excited singlet state 1, $\vec{m}_{1\rightarrow 0}$ and $\vec{\mu}_{1\rightarrow 0}$ the corresponding transition dipole moments for the emissive transition from the lowest excited singlet state back to the ground state, c the speed of light. The sign 'Im' indicates that the imaginary component should be taken. Note that the magnetic dipole operator contains an imaginary number. In the case where the molecular wavefunctions are fully real, the magnetic transition dipole moment will be fully imaginary and the electric dipole moment fully real. In formulae S1 and S2, the magnetic and electric transition dipole moments should be entered in SI units (joule per tesla and coulomb meter). In order to have numbers for m and μ of a convenient magnitude, one could express the magnetic and electric dipole moments for a molecular electronic transition in units of respectively Bohr magneton ($\mu_B = 9.27\times10^{-24}$ joule per tesla) and Debye ($D = 3.34 \times10^{-30}$ coulomb meter). Typical values for the transition dipole moments for a transition that is both electric and magnetic dipole allowed are $\mu = 10$ D and $m = 1.0$ Bohr magneton, which together give a g-value of 0.0037 if the two dipole moments are oriented fully parallel.

For organic dye molecules, the relative contribution of the electric transition dipole to the total dipole strength is usually considerably larger than that of the magnetic dipole contribution ($c^2|\mu|^2 > |m|^2$) so that the expression simplifies to :

$$g_{abs} \cong \frac{4\,\mathrm{Im}\left(\vec{m}_{1\leftarrow 0} \cdot \vec{\mu}_{1\leftarrow 0}\right)}{c\left|\vec{\mu}_{1\leftarrow 0}\right|^2} \quad \text{(S3)}$$

$$g_{lum} \cong \frac{4\,\mathrm{Im}\left(\vec{m}_{1\rightarrow 0} \cdot \vec{\mu}_{1\rightarrow 0}\right)}{c\left|\vec{\mu}_{1\rightarrow 0}\right|^2} \quad \text{(S4)}$$

The strategy that we will use for determining the magnitude of the magnetic transition dipole moment is the following. We start by considering the heptazine core ($C_6H_3N_7$) with D_{3h} symmetry. The lowest excited singlet state of A'$_2$ symmetry is magnetic dipole allowed in the z-direction, but electric dipole forbidden. Given the electric transition dipole moment equal to zero, equations S3 and S4 predict zero circular polarization in absorption and emission, consistent with the achiral nature of the D_{3h} point group. The addition of the enantiopure phenylamide groups makes the molecule *S*-**H** chiral. Yet in dilute solution, *S*-**H** still shows vanishingly small circular dichroism and circular polarization in luminescence, because the stereocenters in the aliphatic side chains are not yet able to enforce helicity in the π-system of the molecule. Stacking of the molecule in the aggregate forces the molecule to adopt a propellor-like shape resulting in a lowering of the symmetry from D_{3h} to D_3.

Table S2. Character Table for the D_{3h} point group.

D_{3h}	E	2 C_3 (z)	3 C'$_2$	σ_h (xy)	2S_3	3 σ_v	rot., transl.	from S_0 via
A'$_1$	1	1	1	1	1	1	-	
A'$_2$	1	1	-1	1	1	-1	R_z	magn. dip.
E'	2	-1	0	2	-1	0	(x,y)	el. dip.
A''$_1$	1	1	1	-1	-1	-1	-	
A''$_2$	1	1	-1	-1	-1	1	z	el. dip.
E''	2	-1	0	-2	1	0	(R_x, R_y)	magn. dip.

Table S3. Character Table for the D_3 point group

D_3	E	2 C_3 (z)	3 C'$_2$	rot., transl.	from S_0 via
A_1	1	1	1	-	
A_2	1	1	-1	R_z, z	magn. + el. dip.
E	2	-1	0	(x,y), (R_x, R_y)	magn. + el. dip.

In the stack, the lowest excited state now has A_2 symmetry and the transition from the ground state to his particular level is now both electric and magnetic dipole allowed in the out-of-plane or z-direction. Because the phenylamide groups only couple weakly to the lowest excited state, we assume that the magnitude of the magnetic transition dipole moment for *S*-**H** in the stack is approximately similar to the magnetic dipole moment in the heptazine core. The phenylamide groups contribute to the electric dipole moment in the z-direction.

To further support the assignment of A2 symmetry to the lowest excited state, we note that the extremely low extinction coefficient for the absorption band is associated with the transition from ground to lowest excited state, the lowest excited state is unlikely to transform according to the E representation. Transitions from the ground state (A_1) to states with E character should be electric dipole allowed with transition dipole vector in the aromatic plane, which is clearly contradicted by the experiment.

So, the lowest excited state transforms as either A_1 or A_2. The transition from the ground state to A_2 is both electric and magnetic dipole allowed in the z-direction (because z and Rz are basis vectors for this representation). A_2 character for the lowest excited state would thus be consistent with the high degree of circular polarization observed experimentally. Also, recent quantum chemical calculations support A_2 character for the lowest excited singlet state in heptazine.[3]

We argue that the lowest excited state of *S*-**H** in the aggregate remains essentially localized on individual molecules because the absorption spectrum in the long wavelength does not show a significant change upon aggregation. We note that this is not the case for the electric dipole allowing optical transitions to the higher excited state of E symmetry located in the spectral region around 330 nm wavelength. Here the absorption spectra clearly show changes upon aggregation (Figure 1). Furthermore, because the transition to the lowest excited state is extremely weak in intensity, interactions between the electric transition dipole moments that normally drive delocalization of the excited states must be very weak. We note that the interaction between two magnetic transition dipoles (of 1 μ_B magnitude) is several orders of magnitude smaller than the interaction between electric transition dipole interactions (of 1 D magnitude) and is thus ineffective in inducing delocalization of the excited state in the stack.[7]

Next, we try to determine the magnitude of the electric transition dipole moment μ for the molecules in the aggregate, because knowing μ and using relations S3 and, S4 one can compute the magnetic transition dipole moment m. We first focus on the determination of the electric transition dipole moment in absorption $\vec{\mu}_{1\leftarrow 0}$.

To determine the electric transition dipole moment μ of the $S_1 \leftarrow S_0$ electronic transition in absorption we make use of the well-known relation between dipole strength $|\mu^2|$ and the molar decadic absorption coefficient ε in solution.[8]

$$|\mu_{1\leftarrow 0}|^2 = \mu^2(S_1 \leftarrow S_0) = \frac{0.00918}{\alpha_{solv}} \times \int_{band} \frac{\varepsilon(\tilde{\nu})}{\tilde{\nu}} d\tilde{\nu} \quad \text{(S5)}$$

Here α is a correction factor for the solvent polarizability. The integral should be taken over the absorption band associated with $S_1 \leftarrow S_0$ electronic transition.

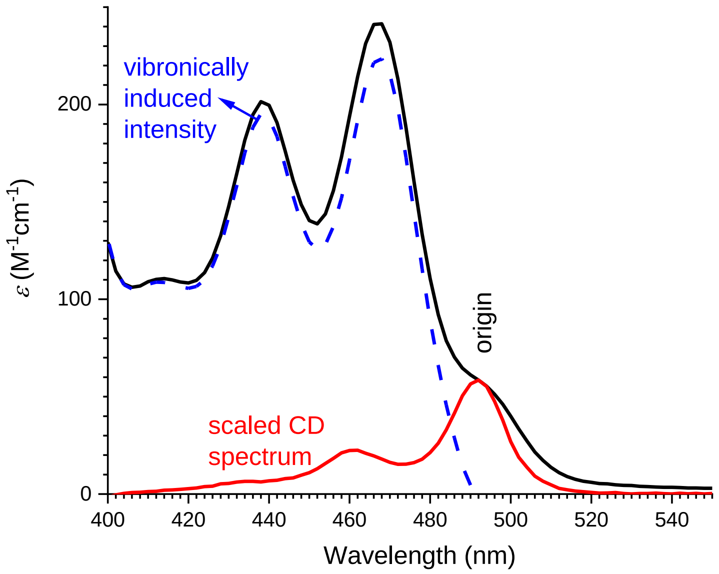

Figure. S17. (Black) Molar absorption coefficient for *S*-**H** in toluene (10 mM). (Red) Scaled CD spectrum of *S*-**H** under the same condition. (Blue) Vibronically induced intensity.

If we now look in detail at the absorption spectrum of the molecule under study, see Figure S17, a complication becomes apparent. The absorption spectrum shows an onset at about 494 nm wavelength with low intensity, followed by maxima of much higher intensity at 467, 438, and 413 nm. From the shoulder at 494 nm, we can identify the 0-0 vibronic band of the transition, i.e. the origin of the transition. This is because the CD spectrum also has its first maximum at 494 nm. The distribution of intensity over the various sub-bands in the absorption spectrum does not follow the standard Franck-Condon profile: the relative intensity of the 0-0 sub-band is way too low. The relatively high intensity of the sub-bands at shorter wavelengths can be accounted for by vibronic mixing. Out-of-plane vibrational modes of *E* symmetry can induce admixture of $\pi\pi^*$ excited states of *E* symmetry to the A_2 lowest excited singlet state. The excited singlet states with *E* symmetry can be reached via electric dipole-allowed transitions from the ground state and are responsible for the strong absorption around 330 nm (See Figure 1). Thus the absorption of heptazine in the 510-410 nm wavelength range has mixed character and contains intrinsic contributions polarized along *z* and vibronically induced component polarized in the *x,y* plane of the molecule.

The induced *x,y* components of the electric transition dipole moment do not contribute to the dipole strength of the 0-0 vibronic transition near 493 nm. Therefore, Eq S3 can be simplified:

$$g_{abs}(493nm) \cong \frac{4Im(m_{1\leftarrow 0,z}\mu_{1\leftarrow 0,z})}{c|\mu_{1\leftarrow 0,z}|^2} \qquad \text{(S5)}$$

The induced x,y components of the electric transition dipole require at least one quantum of the promoting *E* vibrational mode. The energy difference between the maximum of the visible absorption at 467 nm and the origin of the absorption at 493 nm amounts to 1088 cm^{-1}, which is indeed consistent with the highest frequency band for out-of-plane vibrations of the phenylamide heptazine molecule.

Referring back (S5), in order to compute $m_{1\leftarrow 0,z}$ we need to know $\mu_{1\leftarrow 0,z}$. Now to extract $\mu_{1\leftarrow 0,z}$ from experimental data, we make use of the rotational strength of the $S_1\leftarrow S_0$ transition, which is proportional to the integrated circular dichroism of the stacked molecule over the visible band:

$$R_{1\leftarrow 0} = \alpha \int_{band} \frac{\Delta\varepsilon}{\nu} d\nu = Im(\vec{m}_{1\leftarrow 0} \cdot \vec{\mu}_{1\leftarrow 0}) \qquad \text{(S6)}$$

where α is a constant depending on the units used. The rotational strength is related to the dot product of the electric and magnetic transition dipole moments:

$$Im(\vec{m}_{1\leftarrow 0} \cdot \vec{\mu}_{1\leftarrow 0}) \cong Im(m_{1\leftarrow 0,z}\mu_{1\leftarrow 0,z}) = const. \times |\mu_{1\leftarrow 0,z}|^2 \qquad \text{(S7)}$$

where we have assumed that any admixed, *x,y* polarized components of the magnetic transition dipole moment are small compared to the intrinsic magnetic dipole moment of the transition $m_{1\leftarrow 0,z}$. The practical procedure to extract the z-component of the electric dipole moment is now as follows.[9] We take the CD spectrum of aggregated *S*-**H** and scale it such that the maximum of CD signal at 493 nm matches with the absorption at the same wavelength, see Figure S17. The area under the scaled CD spectrum:

$$|\mu_{1\leftarrow 0,z}|^2 = \frac{0.00918}{\alpha_{solv}} \times f_{scale} \times \int_{band} \frac{\Delta\varepsilon(\tilde{\nu})}{\tilde{\nu}} d\tilde{\nu} \qquad \text{(S8)}$$

Where f_{scale} is the dimensionless scaling constant needed to scale the CD spectrum such that its maximum intensity matches the molar absorption at the same wavelength. This procedure, see Figure S17, yields $\mu_{1\leftarrow 0,z} = 0.18\ D$. Using (S3) we then find $m_{1\leftarrow 0,z} = 0.35\mu_B$

14. Determination of the electric and magnetic transition dipole moment of the $S_1\rightarrow S_0$ luminescence band of heptazine.

The Strickler-Berg equation relates the rate of radiative decay of the lowest excited singlet state of a molecule back to the ground state to the absorption spectrum of the corresponding reverse transition from the ground state to the lowest excited state:[10]

$$k_{\text{rad}}^{\text{SB}} = 2.889 \times 10^{-9} n^2 \langle \tilde{\nu}_f^{-3} \rangle^{-1} \int \frac{\varepsilon(\tilde{\nu})}{\tilde{\nu}} d\tilde{\nu} \qquad \text{(S9)}$$

Combining (S9) with (S5) and reverting the microscopic reversibility ($\mu^2(S_1 \rightarrow S_0) = \mu^2(S_1 \leftarrow S_0)$) assumed in the original derivation of (S9), we arrive at:

$$\mu^2(S_1 \rightarrow S_0) = 3.4 \times 10^6 \frac{\langle \tilde{\nu}_f^{-3} \rangle}{\alpha_{solv} n^2} k_{\text{rad}} \quad \text{(S10)}$$

We first determine the rate of radiative decay by measuring the fluorescence decay curve and the fluorescence quantum yield.

When using pure toluene as a solvent, the total concentration of *S*-**H** needs to be quite high (mM) to drive the association to completion. The high concentration leads to problems with self-absorption in the fluorescence measurements while at low concentration, unpolarized fluorescence from not aggregated molecules dominates fluorescence.

In the solvent mixture of toluene and methylcyclohexane, the molecules can be quantitatively aggregated at a lower total concentration, and the experimental difficulties mentioned above can be avoided.

In toluene/MCH (1/3 vol/vol), the averaged excited state lifetime amounts to 61 ns with a fluorescence quantum yield of 5.8 %. This yields a radiative decay constant of k_{rad}= 0.00095 ns^{-1}. Next, we realize that radiative decay can also occur due to the vibronic admixture of dipole transitions polarized in the plane of the molecule.

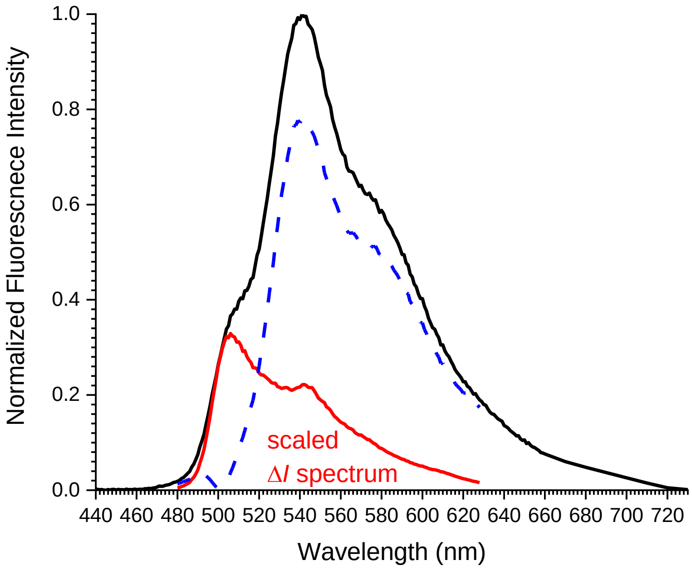

Figure S18. Decomposition of the fluorescence of *S*-**H** in Tol/MCH into components polarized along the molecular *z*-axis, (red line) and components polarized in the molecular *x,y*-plane (dashed blue line)

To accomplish this decomposition, we apply a procedure similar to the one used for decomposing the absorption spectrum (see above). From the measurement of g_{lum} (see Figure S18), we compute the band profile of the circular differential emission ($\Delta I = I_L - I_R$). We scale the ΔI spectrum such that it matches the fluorescence spectrum in intensity at the onset of the band near 495 nm, see Figure S18. We then integrate both the total and the scale ΔI spectrum and find that only 25 % of the radiative decay is due to transitions polarized parallel to the molecule *z*-axis. Incidentally for the energy difference between the maximum of the DI spectrum and the maximum of the total emission, we find the frequency of the promoting out-of-plane vibrational mode with E symmetry is around 1280 cm^{-1}, largely consistent with the estimate from the absorption spectrum. We note that these frequencies do not need to match exactly because they refer to ground and excited state out-of-plan vibrations.

Finally using relation S10, we find that the z-component of transition dipole in emission is $\mu_{1\rightarrow 0,z} = 0.20\ D$. Taking the maximum dissymmetry ratio in emission $g_{\text{lum}} = -0.068$ we obtain the magnetic transition dipole moment in emission $m_{0\rightarrow 1,z} = 0.36\ \mu_B$

15. Calculation of the magnetic transition dipole moment for the heptazine core molecule.

In this section, we provide a simple quantum chemical calculation of the magnetic transition dipole moment of the heptazine moiety ($C_6H_3N_7$ 1,3,4,6,7,9,9b-Heptaazaphenalene) with D_{3h} symmetry. The molecular geometry is known from X-ray crystallography.[11] In our calculation, we aim at maximal transparency and traceability rather than quantum chemical accuracy. The main reason for this is to avoid any possible confusion associated with the use of units from different systems (SI, CGS, or atomic). We focus on the p-electron system and neglect any involvement of electrons in s-orbitals.

The magnetic transition dipole moment of a transition between singlet states is obtained from the evaluation of the matrix element of the orbital angular momentum operator *L:*

$$\vec{m}_{fi} = \langle f|\hat{\vec{L}}|i\rangle \frac{\mu_B}{\hbar} \tag{S11}$$

Here the values of the matrix elements of the orbital angular momentum operator in units of $\hbar$ are equal to the values of the magnetic transition dipole moment in units of Bohr magneton.

Considering the three-fold rotational symmetry of heptazine around the z-axis and the fact that the molecule has only 6 carbon and 6 nitrogen atoms in its outer rim, it follows that any p-orbital cannot have more than 3 vertical nodal planes. The Lz angular momentum quantum number of a p electron can therefore not exceed 3 units of $\hbar$ in magnitude. The magnetic transition dipole moment must be smaller than 3 Bohr magnetons.

For particles confined to a perfectly circular orbit in the *x*,*y*-plane of fixed radius R the states with Lz =+3$\hbar$ and -3$\hbar$ are

$$\psi_+(\phi) = \frac{1}{\sqrt{2\pi}} e^{+i3\phi}$$
$$\psi_-(\phi) = \frac{1}{\sqrt{2\pi}} e^{-i3\phi} \tag{S12}$$

From these degenerate states, we can construct two fully real states:

$$\psi_p(\phi) = \frac{1}{\sqrt{2}}\left(\psi_+(\phi) + \psi_-(\phi)\right)$$
$$\psi_m(\phi) = \frac{1}{\sqrt{2}}\left(\psi_+(\phi) - \psi_-(\phi)\right) \tag{S13}$$

Then expressing the angular momentum operator in terms of the angle ϕ

$$\hat{L}_z = -i\hbar \frac{d}{d\phi} \tag{S14}$$

, one finds that the maximal magnetic dipole moment is achieved for a transition of an electron from the ψ_p to the ψ_m state because:

$$\hat{L}_z \psi_m = (-i\hbar) 3 \psi_p \tag{S15}$$

And so:

$$\left|\langle \psi_p|\hat{L}_z|\psi_m\rangle\right| = 3\left|\langle \psi_p|\psi_p\rangle\right| = 3\hbar \tag{S16}$$

The idealized ψ_p to the ψ_m state allow us to build an intuitive picture of which pair of orbitals will give the maximum magnetic dipole moment when an electron is transferred between these two orbitals via optical excitation. We first plot the states ψ_p and ψ_m as function of the angle ϕ in the range (0, 2π), see Figure S19.

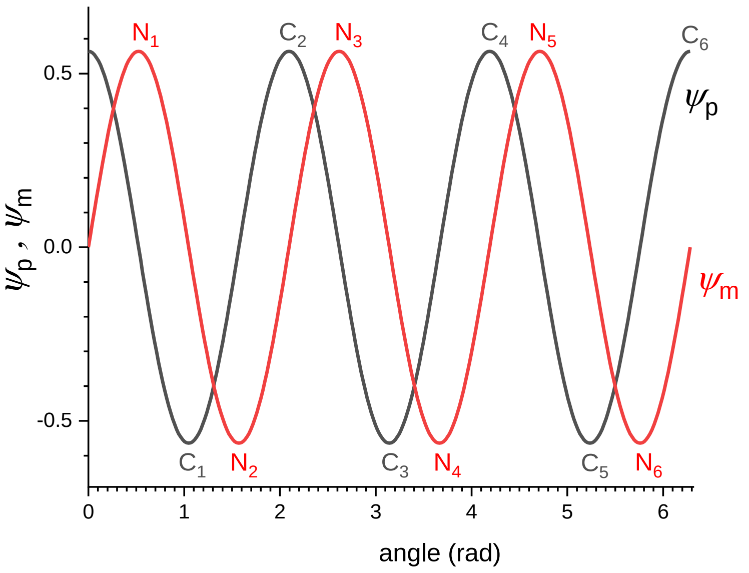

Figure S19. Quantum states ψ_p and ψ_m for an electron on a ring.

Next we indicate the approximate position of the 6 nitrogen and six carbon atoms in the outer ring (N1..N6 and C1..C6), see again Figure S19. We note that ψ_p has six nodes at exactly the position of the nitrogen atoms and that ψ_m also has six nodes but now at the place where the nitrogen atoms are positioned. It then follows that the maximum magnetic dipole moment is expected for an optical transition of an electron from a molecular orbital with three vertical nodal planes including all 6 nitrogen p_z atomic orbitals to a molecular orbital with also three vertical nodal planes but now with contributions from only the six p_z atoms orbitals on the carbon atoms in the outer ring. Finally, if we now look at the HOMO and LUMO orbitals in Figure 3c we see that indeed these each have three vertical nodal planes with the homo localized on nitrogen and the LUMO localized largely on carbon. Thus, for the HOMO-LUMO excitation of the heptazine moiety, we expect a large transition dipole moment with magnitude < 3 Bohr magneton. To get a better estimate of the magnetic transition dipole moment we perform Hückel molecular orbital calculations. As a basis set, we take the 13 atomic p_z orbitals of the carbon and nitrogen atoms. The Hamiltonian we express as:

$$\hat{H} = \begin{bmatrix} \alpha & \beta & 0 & 0 & 0 & 0 & 0 & 0 & 0 & 0 & 0 & \beta & 0 \\ \beta & -\alpha & \beta & 0 & 0 & 0 & 0 & 0 & 0 & 0 & 0 & 0 & 0 \\ 0 & \beta & \alpha & \beta & 0 & 0 & 0 & 0 & 0 & 0 & 0 & 0 & 0 \\ 0 & 0 & \beta & -\alpha & \beta & 0 & 0 & 0 & 0 & 0 & 0 & 0 & \beta_L \\ 0 & 0 & 0 & \beta & \alpha & \beta & 0 & 0 & 0 & 0 & 0 & 0 & 0 \\ 0 & 0 & 0 & 0 & \beta & -\alpha & \beta & 0 & 0 & 0 & 0 & 0 & 0 \\ 0 & 0 & 0 & 0 & 0 & \beta & \alpha & \beta & 0 & 0 & 0 & 0 & 0 \\ 0 & 0 & 0 & 0 & 0 & 0 & \beta & -\alpha & \beta & 0 & 0 & 0 & \beta_L \\ 0 & 0 & 0 & 0 & 0 & 0 & 0 & \beta & \alpha & \beta & 0 & 0 & 0 \\ 0 & 0 & 0 & 0 & 0 & 0 & 0 & 0 & \beta & -\alpha & \beta & 0 & 0 \\ 0 & 0 & 0 & 0 & 0 & 0 & 0 & 0 & 0 & \beta & \alpha & \beta & 0 \\ \beta & 0 & 0 & 0 & 0 & 0 & 0 & 0 & 0 & 0 & \beta & -\alpha & \beta_L \\ 0 & 0 & 0 & \beta_L & 0 & 0 & 0 & \beta_L & 0 & 0 & 0 & \beta_L & \alpha \end{bmatrix} \quad \text{(S17)}$$

Where we use $\alpha = -1$ eV; $\beta = -3$ eV and $\beta_L = -1.5$. The smaller absolute magnitude of β_L versus β reflects the significantly longer bond length between the center nitrogen and the carbon atoms (1.38 Å) versus the C-N bond length in the outer rim (1.31-1.34 Å). The eigenfunctions of the Hamiltonian in (S17) indeed feature a HOMO orbital localized on the outer 6 nitrogen atoms with three vertical nodal planes of A"$_1$ symmetry. The LUMO with A"$_2$ symmetry, is largely centered on the carbon atoms, and also has three vertical nodal planes.

To calculate the magnetic transition dipole moment, we express the atomic p_z orbitals as:

$$\varphi_{2p} = \frac{1}{\sqrt{N}} z\, exp\left[-\frac{Z_{eff,2p} r}{2a_0}\right] \quad \text{(S18)}$$

With z the height above the aromatic plane, r the distance to the center of the orbital and $Z_{\text{eff,2p}}$ the effective nuclear charge for the 2p orbital[12] (C : $Z_{\text{eff,2p}} = 3.25$ and N: $Z_{\text{eff,2p}} = 3.9$). The final of the magnetic transition dipole moment of the HOMO to LUMO excitation then involves incorporation of the orbital coefficients, expressing z and r in polar coordinates, appropriate normalization of the orbital with polar coordinates, and finally numerical integration of the matrix element.

$$\left|m_{HOMO\to LUMO,z}\right| = \frac{\mu_B}{h}\left|\langle HOMO|\hat{L}_z|LUMO\rangle\right| \quad \text{(S19)}$$

The final result is $\left|m_{HOMO\to LUMO,z}\right| = 0.4\,\mu_B$, *i.e.* a magnetic transition dipole moment of 0.4 bohr magneton.

16. DFT calculation.

The electronic structures of heptazine (*S*-**H**) were calculated using time-dependent density functional theory (TDDFT) with the three-parameter Becke−Lee−Yang−Parr hybrid density functional (hybrid-B3LYP) using AMSjobs 2022.1 program. To simplify the calculation, aliphatic chains on the molecule were not involved in the simulations due to their limited effect on the electronic structure of heptazine. The optimized structure of *S*-**H** from DFT calculation indicated a flat core of heptazine, while the rotation angle of the phenol group to the heptazine plane is less than 10°. Moreover, the dihedral angle of the carbonyl group and phenyl group is 31°, while the other two dihedral angles are around 10°.

17. IR and NMR data.

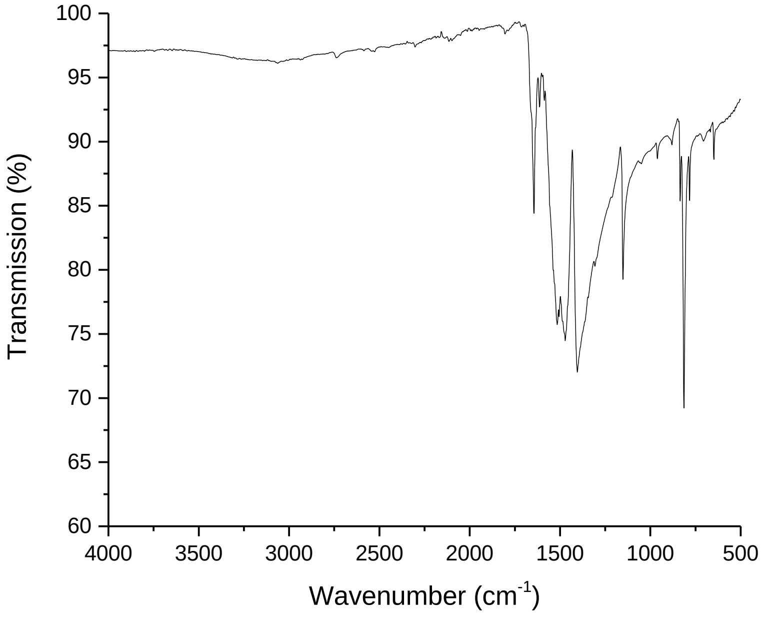

Figure S20. IR spectrum of Potassium Cyamelurate (**2**).

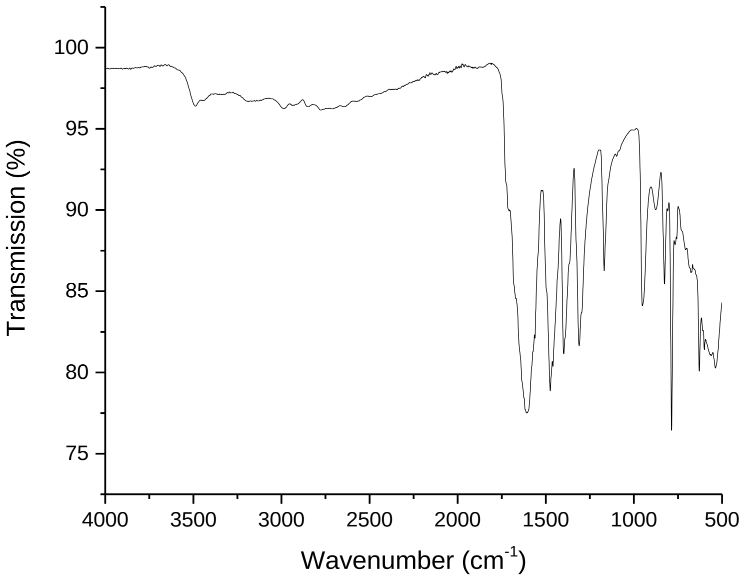

Figure S21. IR spectrum of Cyameluric acid (**3**).

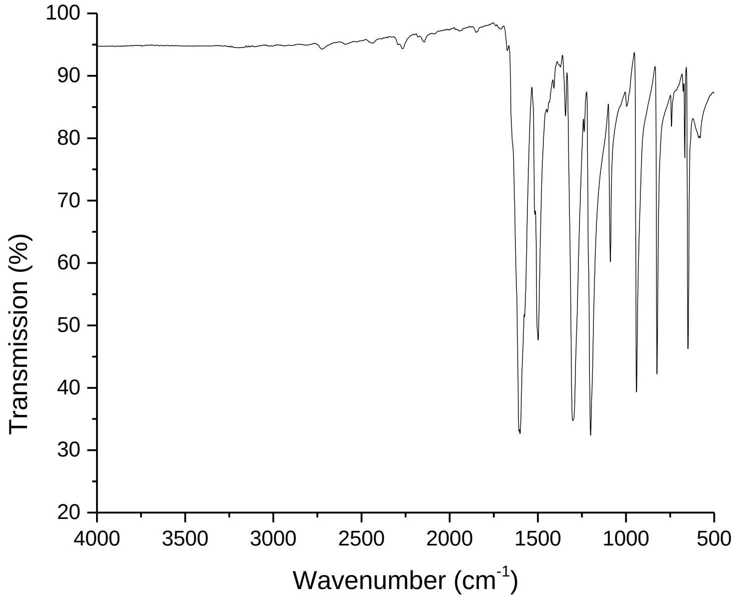

Figure S22. IR spectrum of Cyameluric Chloride (**4**).

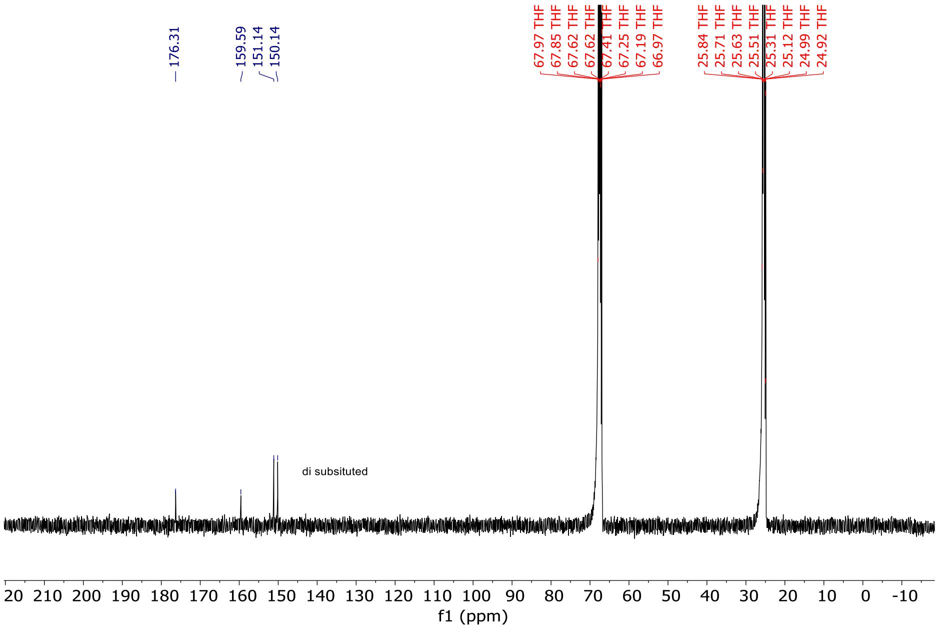

Figure S23. ^{13}C NMR (100 MHz, THF-d_8) spectrum of Cyameluric chloride (**4**).

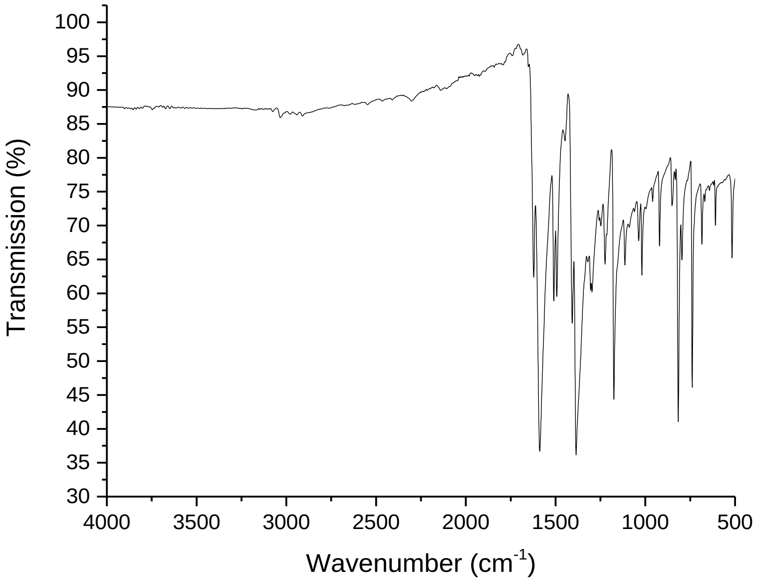

Figure S24. IR spectrum of 2,5,8-tri-p-tolyl-1,3,3a1,4,6,7,9-Heptaazaphenalene (**5**).

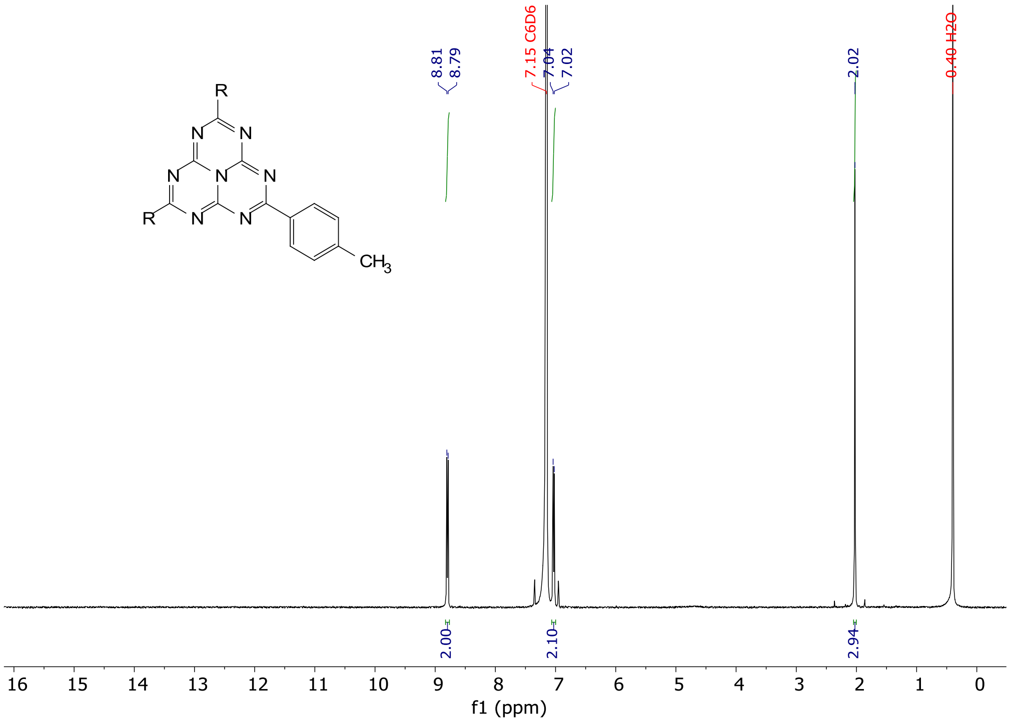

Figure S25. ^{1}H NMR (400 MHz, C_6D_6) spectrum of 2,5,8-tri-p-tolyl-1,3,3a1,4,6,7,9-Heptaazaphenalene (**5**).

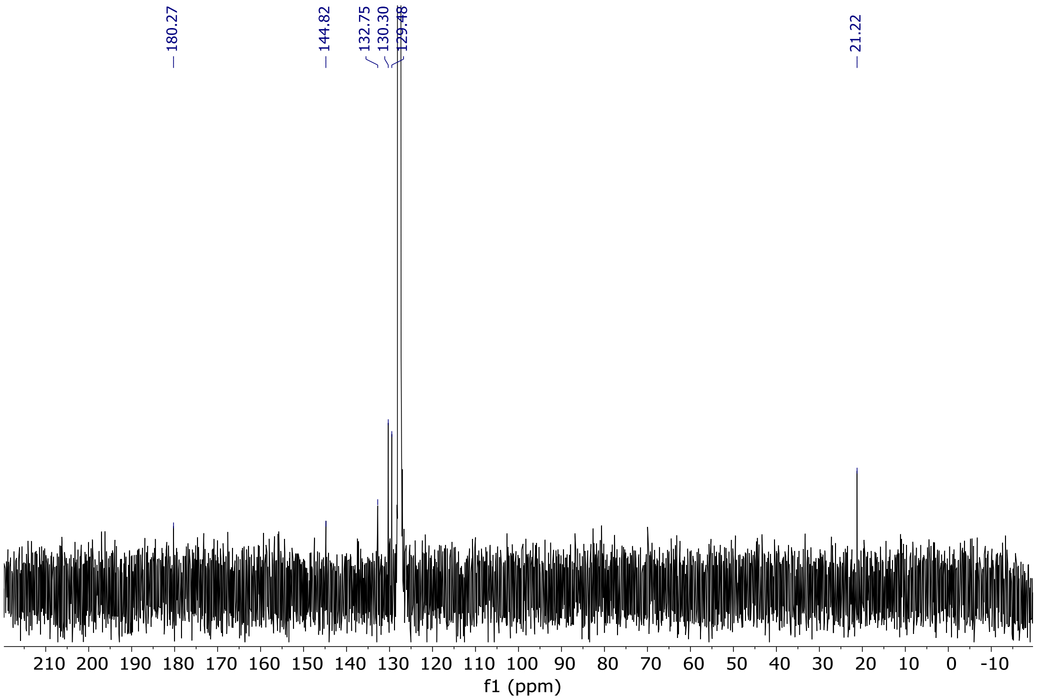

Figure S26. ^{13}C NMR (100 MHz, C_6D_6) spectrum of 2,5,8-tri-p-tolyl-1,3,3a1,4,6,7,9-Heptaazaphenalene (**5**).

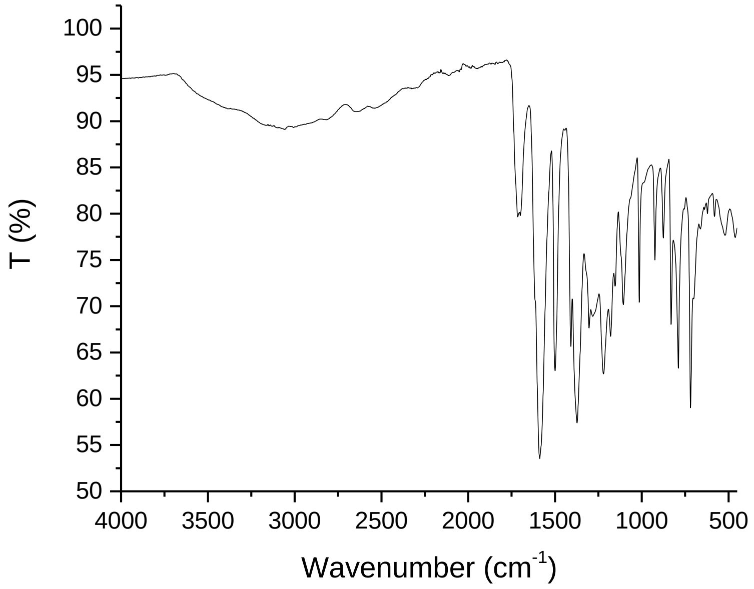

Figure S27. IR spectrum of 4,4',4''-(1,3,3a1,4,6,7,9-Heptaazaphenalene-2,5,8-triyl)tribenzoic acid (**6**).

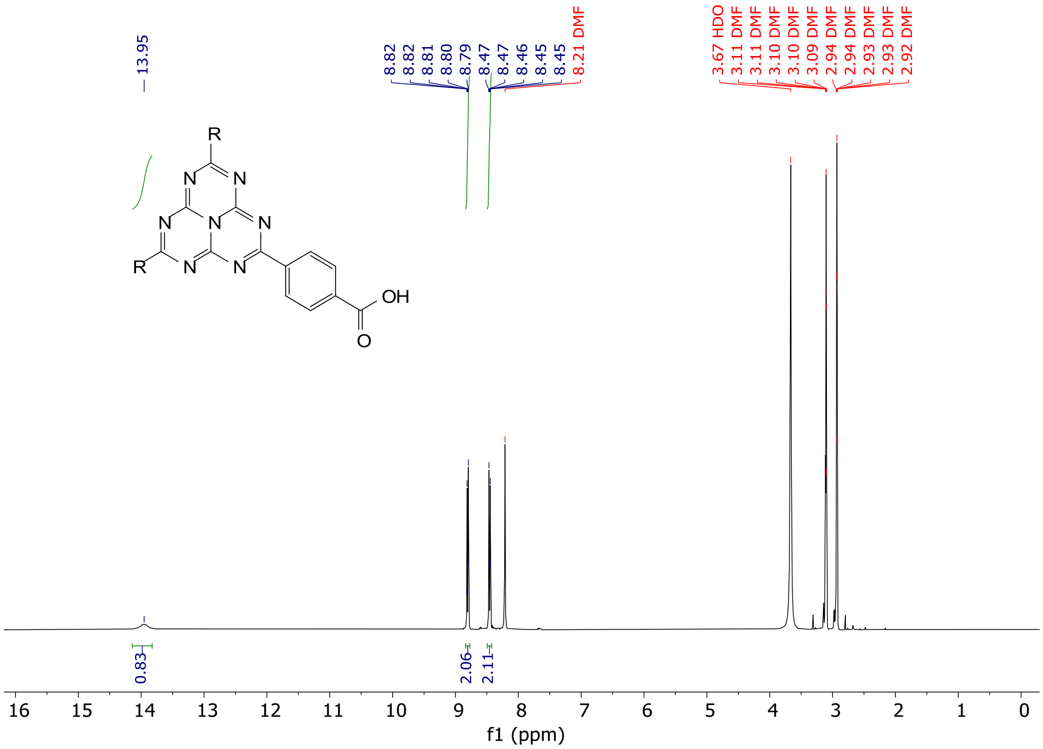

Figure S28. ^{1}H NMR spectrum (400 MHz, DMF-d_7) of 4,4',4''-(1,3,3a1,4,6,7,9-Heptaazaphenalene-2,5,8-triyl)tribenzoic acid (**6**).

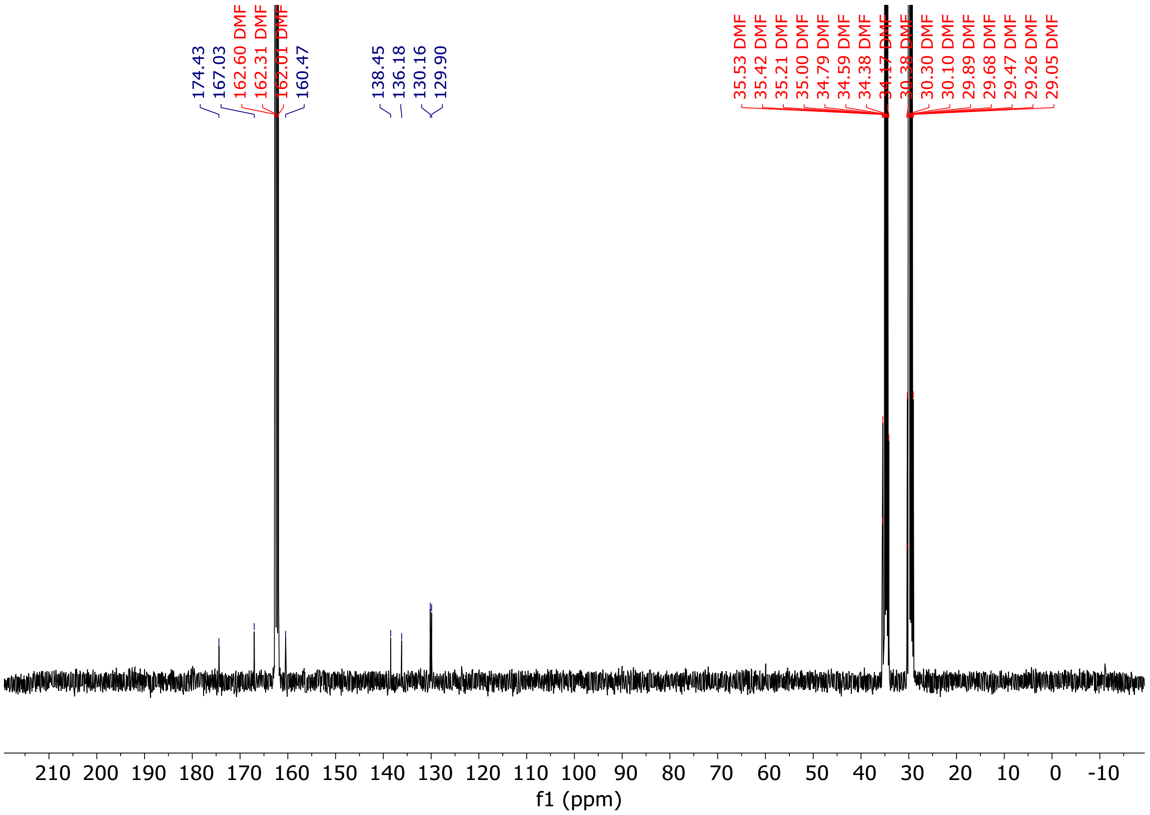

Figure S29. ^{13}C NMR (100 MHz, DMF-d_7) spectrum of 4,4',4''-(1,3,3a1,4,6,7,9-Heptaazaphenalene-2,5,8-triyl)tribenzoic acid (**6**).

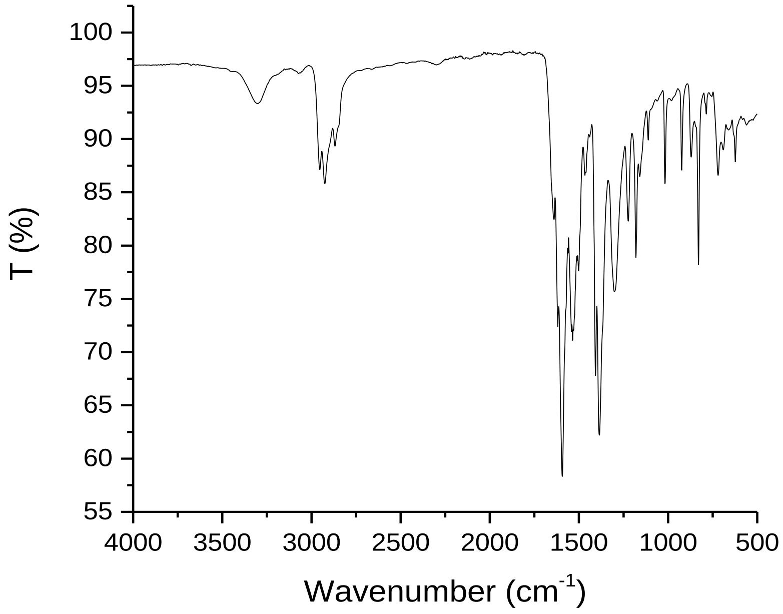

Figure S30. IR spectrum of 4,4',4''-(1,3,3a1,4,6,7,9-Heptaazaphenalene-2,5,8-triyl)tris(N-((*S*)-3,7-dimethyloctyl)benzamide)(***S*-H**)

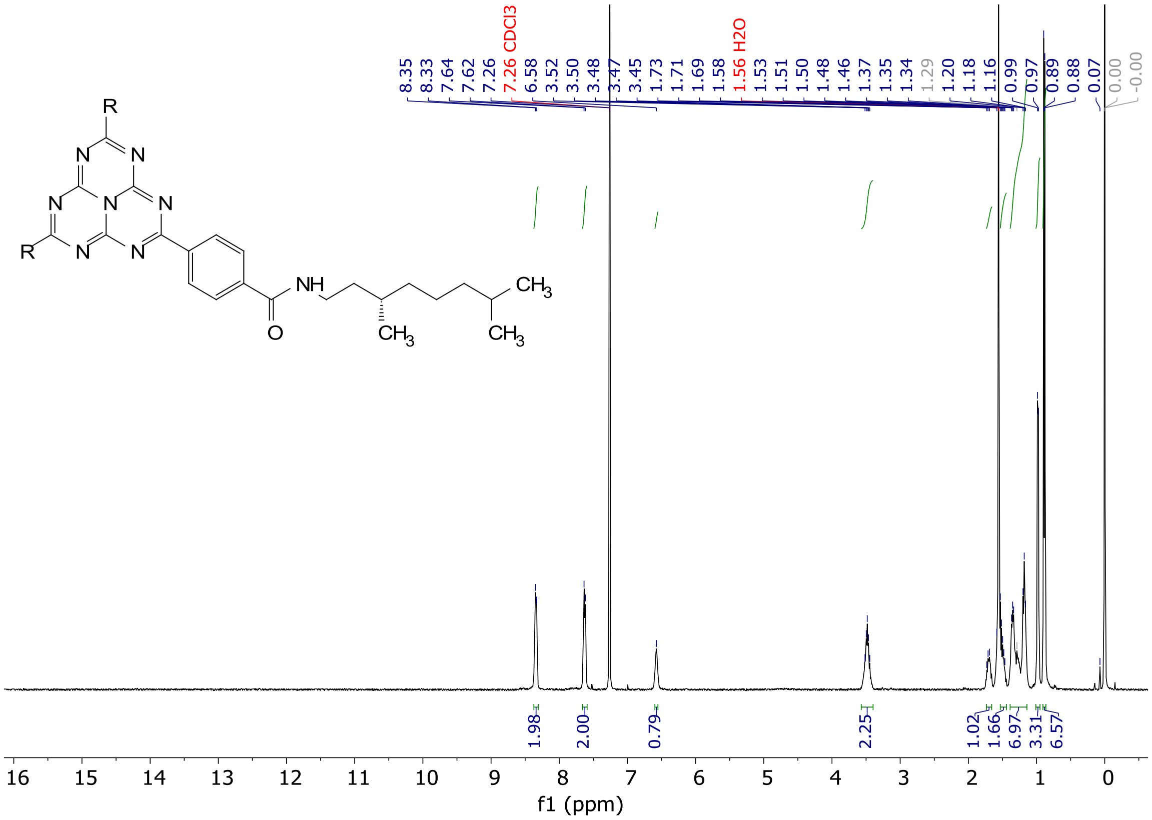

Figure S31. ^{1}H NMR (400 MHz, $CDCl_3$) spectrum of 4,4',4"-(1,3,3a1,4,6,7,9-Heptaazaphenalene-2,5,8-triyl)tris(N-((*S*)-3,7-dimethyloctyl)benzamide)(*S*-**H**) (2mg mL^{-1}). We note that *S*-**H** forms aggregates at 2mg mL^{-1} in $CDCl_3$. At lower concentrations, signals of aromatic protons shifted downfield, while the signal of N-H shifted upfield.

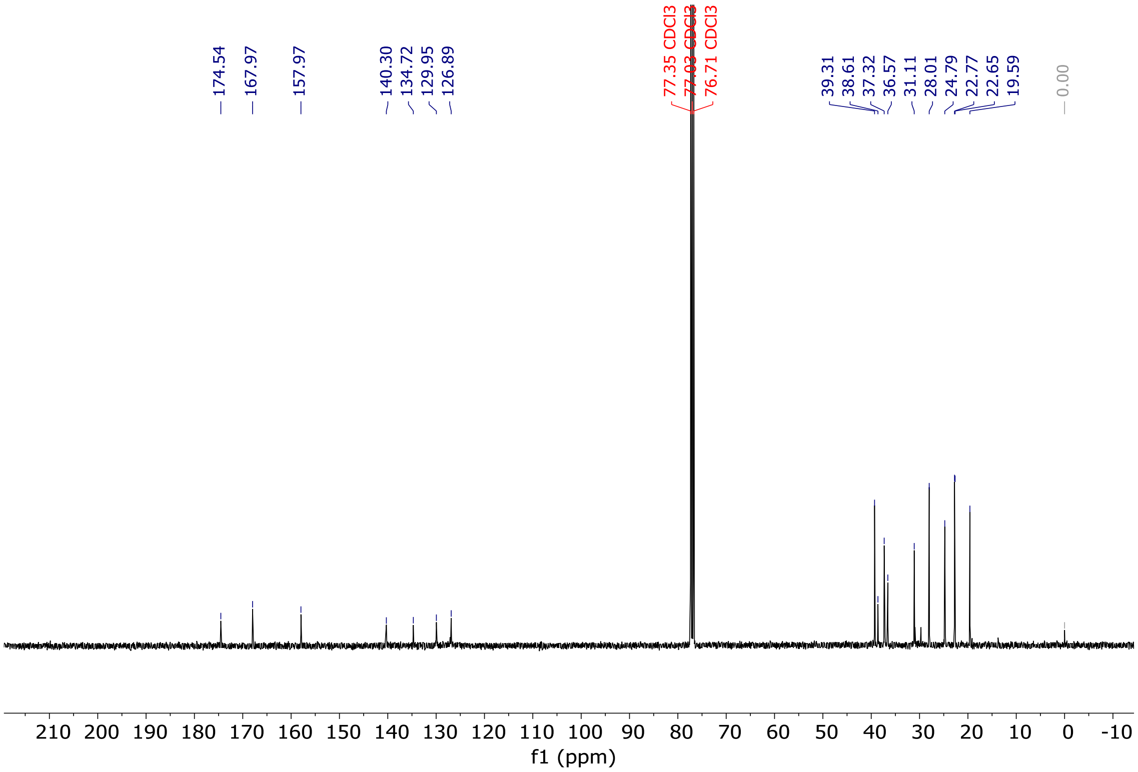

Figure S32. ^{13}C NMR (100 MHz, $CDCl_3$) spectrum of 4,4',4"-(1,3,3a1,4,6,7,9-Heptaazaphenalene-2,5,8-triyl)tris(N-((*S*)-3,7-dimethyloctyl)benzamide) (***S*-H**).

References:

1 Nucleation–elongation: a mechanism for cooperative supramolecular polymerization. Zhao, D.; Moore, J.S. *Org. Biomol. Chem.* **2003**, *1*, 3471-3491.

2 Competing Interactions in Hierarchical Porphyrin Self-Assembly Introduce Robustness in Pathway Complexity. Mabesoone, M.F.J.; Markvoort, A.J. ; Banno, M. ; Yamaguchi, T. ; Helmich, F.; Naito, Y. ; Yashima, E.; Palmans, A.R.A.; Meijer, E.W. *J. Am. Chem. Soc.* **2018**, *140*, 7810-7819.

3 Triangular boron carbon nitrides: an unexplored family of chromophores with unique properties for photocatalysis and optoelectronics. Pios, S.; Huang, X.; Sobolewski, A. L.; Domcke, W. *Phys. Chem. Chem. Phys.* **2021**, *23*, 12968-12975.

4 Solar Energy Harvesting with Carbon Nitrides and N-Heterocyclic Frameworks: Do We Understand the Mechanism? Domcke, W.; Ehrmaier, J. ;Sobolewski, A. L. *ChemPhotoChem* 2019, *3*, 10-23.

5 Photooxidation of water with heptazine-based molecular photocatalysts: Insights from spectroscopy and computational chemistry. Domcke, W.; Sobolewski, A. L.; Schlenker, C. W. *J. Chem. Phys*. **2020**, *153*, 100902.

6 a) Optical Frequency Magnetic Dipole Transitions. Karaveli, S.; Zia R. In: Bhushan, B. (ed) Encyclopedia of Nanotechnology, 2012, Springer, Dordrecht. b) Excitation of Magnetic Dipole Transitions at Optical Frequencies. Kasperczyk, M.; Person, S.; Ananias, D.; Carlos, L. D.; Novotny, L. *Phys. Rev. Lett.* **2015**, *114*, 163903. c) Linearly polarized luminescence spectra of Eu(2,6-pyridine-dicarboxylate)$_3^{3-}$ in hydroxylic solution. Meskers, S. C.J.; Riehl, J. P.; Dekkers, H. P.J.M. *Chem. Phys. Lett.* **1993**, *216*, 241-246.

7 Symmetry Rules for Optical Rotation. Schellman, J.A. *Acc. Chem. Res.* **1968**, *1*, 144-151.

8 Dipole and Oscillator Strengths of Chromophores in Solution. Knox, R.S., *Photochem.Photobiol.* **2003**, *77*, 492-496.

9 The n-π* Absorption and Emission of Optically Active trans-β-Hydrindanone and trans-β-Thiohydrindanone. Emeis, C.A.; Oosterhoff, L.J. *J. Chem. Phys.* **1971**, *54*, 4809-4819.

10 Relationship between absorption intensity and fluorescent lifetime of molecules. Strickler, S. J.; Berg, R. A. *J. Chem. Phys*. **1962**, *37*, 814-822.

11 Synthesis and structure of tri-s-triazine. Hosmane, R.S.; Rossman, M.A.; Leonard, N.J. *J. Am. Chem. Soc.* **1982**, *104*, 5497–5499.

12 Screening Percentages Based on Slater Effective Nuclear Charge as a Versatile Tool for Teaching Periodic Trends. Waldron, K. A.; Fehringer, E. M.; Streeb, A.E.; Trosky, J.E.; Pearson, J. J. *J. Chem. Educ.* **2001**, *78*, 567-694.
